# Animal Models: A Useful Tool to Unveil Metabolic Changes in Hepatocellular Carcinoma

**DOI:** 10.3390/cancers12113318

**Published:** 2020-11-10

**Authors:** Marina Serra, Amedeo Columbano, Andrea Perra, Marta Anna Kowalik

**Affiliations:** Unit of Oncology and Molecular Pathology, Department of Biomedical Sciences, University of Cagliari, 09042 Monserrato, Italy; marina.serra@unica.it (M.S.); columbano@unica.it (A.C.)

**Keywords:** HCC, glycolysis, OXPHOS, PPP, miRNA

## Abstract

**Simple Summary:**

Hepatocellular carcinoma (HCC) represents an important health problem. At the moment, systemic therapies offered only modest clinical benefits. Thus, HCC represents a cancer extremely difficult to treat, and therapeutic breakthroughs are urgently needed. Metabolic reprogramming of neoplastic cells has been recognized as one of the core hallmarks of cancer. Experimental animal models represent an important tool that allows to investigate metabolic changes underlying HCC development and progression. In the present review, we characterize available rodent models of hepatocarcinogenesis. Moreover, we discuss the possibility that pharmacological targeting of Warburg metabolism may represent an additional tool to improve already available therapeutic approaches for HCC.

**Abstract:**

Hepatocellular carcinoma (HCC) is one the most frequent and lethal human cancers. At present, no effective treatment for advanced HCC exist; therefore, the overall prognosis for HCC patients remains dismal. In recent years, a better knowledge of the signaling pathways involved in the regulation of HCC development and progression, has led to the identification of novel potential targets for therapeutic strategies. However, the obtained benefits from current therapeutic options are disappointing. Altered cancer metabolism has become a topic of renewed interest in the last decades, and it has been included among the core hallmarks of cancer. In the light of growing evidence for metabolic reprogramming in cancer, a wide number of experimental animal models have been exploited to study metabolic changes characterizing HCC development and progression and to further expand our knowledge of this tumor. In the present review, we discuss several rodent models of hepatocarcinogenesis, that contributed to elucidate the metabolic profile of HCC and the implications of these changes in modulating the aggressiveness of neoplastic cells. We also highlight the apparently contrasting results stemming from different animal models. Finally, we analyze whether these observations could be exploited to improve current therapeutic strategies for HCC.

## 1. Introduction

### 1.1. Hepatocellular Carcinoma (HCC)

Primary liver cancer, which includes predominantly hepatocellular carcinoma (HCC) (75–85% of cases), ranks third in terms of mortality worldwide, and represents an important challenge for global health [1]. Regions with high or low-rate incidence of HCC are characterized by a prevalence of different risk factors. Importantly, most HCCs develop in patients with underlying liver cirrhosis and are associated with different etiologies, including hepatitis B virus (HBV) or hepatitis C virus (HCV) chronic infection, exposure to carcinogenic compounds like aflatoxin B1, alcoholic and non-alcoholic fatty liver disease (AFLD and NAFLD), smoking and type 2 diabetes [2]. 

Presently, there are only a few drugs approved by Food and Drug Administration (FDA). The first is Sorafenib, an oral multikinase inhibitor of the vascular endothelial growth factor receptor (VEGRF), the platelet-derived growth factor receptor (PDGFR) and Raf for the treatment of unresectable HCC. However, an increased survival of only 3 months was shown in the Sorafenib Hepatocellular Carcinoma Assessment Randomized Protocol (SHARP) among patients with advanced disease [3]. A similar slight improvement was obtained when sorafenib treatment was followed by regorafenib, another tyrosine kinase (TK) inhibitor [4]. New therapeutic strategies, such as Atezolizumab plus Bevacizumab for advanced and metastatic HCC together with Lenvatinib as the frontline line treatment option, followed by Cabozantinib and Ramucirumab as the second line targeted agents have been recently introduced [5,6]. In addition, immunotherapy has been proposed for HCC treatment, based on the finding that the programmed cell death protein 1 (PD-1) and programmed death-ligand 1 (PD-L1) axis is an adaptive immune resistance mechanism used by cancer cells to overcome immune anti-tumor activity. Several studies have demonstrated that overexpression of PD-L1 is associated with more aggressive phenotype and worse prognosis of HCC [7,8,9]. Accordingly, nivolumab, an inhibitor of PD-1 exhibited positive results in patients with advanced HCC with or without chronic viral hepatitis [10], although the overall therapeutic effect remains still unsatisfactory. However, the results are not satisfactory, and no effective systemic therapy exists for patients with advanced HCC.

A more complete understanding of the molecular mechanisms involved in HCC development and progression thus is required to improve therapeutic strategies against this lethal cancer. Over the last years, many fundamental studies have explored genomic alterations and identified the most frequently mutated genes in HCC. Mutations of the *TERT* promoter able to activate telomerase expression, have been described as the earliest alterations in HCC development, as they were found in a significant percentage of dysplastic nodules [11], while molecular alterations in cell cycle control (*TP53*, *RB1*, *CCND1*, *CDKN2A*), Wnt/β-catenin signaling (*CTNNB1*, *AXIN1*), oxidative stress response (*NFE2L2*, *KEAP1*), epigenetic regulation (*ARID1A*, *ARID2*) and the protein kinase B/mammalian target of rapamycin (AKT/mTOR) and mitogen-activated protein (MAP) kinase pathway were shown to take place at late stages of tumorigenesis [12,13,14,15,16]. More recently, epigenetic events, such as methylation status, have been argued to play a critical role in HCC progression [17].

### 1.2. Metabolic Changes Observed in Human HCC

Oncogenic mutations cause alterations to numerous signaling pathways that affect tumor cell metabolism and lead to enhanced survival and growth [18,19]. Parallel, though limited, studies have investigated the presence and possible significance of metabolic alterations in HCC, engendering new insight into this cancer type. Worth of reminding is that a marked interest in tumor metabolism has been observed since almost a century, when aerobic glycolysis or ‘Warburg phenomenon/effect’ [20,21] was described. This metabolic shift, reporting that most cancer cells enhance glucose utilization independently of oxygen availability, has now achieved the status of a core hallmark of cancer [22]. The switch from oxidative phosphorylation (OXPHOS)—the most efficient pathway for producing adenosine triphosphate (ATP) to glycolysis observed in cancer cells is likely required to withstand the increased anabolic demands of tumor cells [23,24]. Such needs of cancer cells are satisfied mainly by conveying metabolites into the pentose phosphate pathway (PPP). PPP provides both ribose-5-phosphate, a structural component of nucleotides, to sustain cell proliferation and reduced nicotinamide adenine dinucleotide phosphate (NADPH), utilized for fatty acid and cholesterol biosynthesis, as well as required for the generation of reduced glutathione (GSH), a major scavenger of reactive oxygen species. These processes are regulated primarily by the action of glucose-6-phosphate dehydrogenase (G6PD), the rate-limiting enzyme of the PPP [25,26,27]. 

The metabolic switch from OXPHOS to glycolysis allows neoplastic cells to enhance their biosynthetic capabilities by expressing a tumor-specific form of pyruvate kinase (PK). This catalyzes the rate-limiting, ATP-generating, irreversible reaction of glycolysis, the conversion of phosphoenolpyruvate (PEP) to pyruvate [28,29,30]. The M2 isoform of pyruvate kinase (PKM2) of cancer cells is under control of numerous allosteric modulators that can activate or inhibit its activity [28,29] and a switch between a highly active tetrameric form of PKM2 and an almost inactive dimeric form is considered as one of the main regulators of Warburg metabolism [31]. Inhibition of PKM2 can also increase levels of glucose-6-phosphate in order to increase oxidative PPP flux [32].

With regard to HCC, different aspects of metabolic reprogramming have been investigated over the last years [33]. Firstly, metabolomics, which summarizes the metabolic status of the organism and reflects the dynamic interactions between genes, proteins and the environment [34,35], showed an alteration of energy metabolism between HCC and adjacent non-involved tissues [36]. Successively, combined transcriptomic and metabolomic studies of energy metabolism in HCC demonstrated an increase in glycolysis over mitochondrial OXPHOS [37], as also recently reported [16]. In particular, OXPHOS was shown to be among the most significantly downregulated pathways in a homogeneous HCC subgroup driven by the activation of cyclin A2 (CCNA2) or cyclin E1 (CCNE1) gene [38]. A recent thorough study of the expression of metabolic genes in human HCCs [39] reported more than 600 consistently altered metabolic genes, mostly involved in glycolysis, PPP, nucleotide biosynthesis, tricarboxylic acid (TCA) cycle, OXPHOS, glucose and glutamine transporters, as well as lipid biochemistry. Many of the identified metabolic genes correlated with progression markers or were predictive of overall survival outcome [39]. 

With regard to the individual players of aerobic glycolysis, much attention has been paid to the family of glucose transporters (GLUT), consisting of 14 subtypes of glucose transporters in humans [40]. In particular, GLUT1, the key determinant of glucose uptake, resulted to be often aberrantly expressed in different cancer types. Accordingly, elevated *GLUT1* expression levels were associated with a higher proliferation, advanced tumor stage and poor differentiation in HCC tissues when compared with matched non-tumor tissue [41,42]. Another member of GLUT family, that caught the attention in HCC was GLUT2. Several studies reported that higher *GLUT2* expression was indicative of poor patient survival [43,44]. However, Kim et al. [45] found that the prognostic value of *GLUT2* was more significant in those patients who did not present major risk factors for HCC, such as alcohol consumption and HBV/HCV infections. Very recently, Gao et al. [46] elucidated the correlation between *GLUT3* expression in HCC tissues and the clinicopathological features. Interestingly, increased *GLUT3* expression in HCC tissues was significantly associated with elevated α-fetoprotein levels (AFP), poor differentiation and Tumor-Node-Metastasis stages III and IV; it also correlated with reduced overall survival of patients.

Apart from glucose transporters, hexokinases that catalyze the first key step of glycolysis in which glucose is phosphorylated into glucose 6-phosphate, are recognized as the central players in the regulation of glycolysis [47]. While normal differentiated adult hepatocytes exhibit glucokinase (GCK, HK4) for the regulation of glucose homeostasis, HCC cells are metabolically distinct from normal hepatocytes [47]. There is a switch from HK4 to the high-affinity hexokinase-2 (HK2). Repressing glucokinase and expressing HK2 isoform by cancer cells allows the high glycolytic rates that occur in anaerobic metabolism in cancer development [48,49]. Higher levels of HK2, which plays a critical role in initiating and maintaining the enhanced glucose catabolic rates of rapidly growing tumors [47,48], were observed in liver cell change/dysplasia in cirrhosis (LCD) and HCCs when compared with non-dysplastic cirrhosis (NDC), indicating a crucial role of HK2 in the onset of HCC [50]. Higher expression of HK2 was associated with more aggressive histological features [50], worse overall survival and poor prognosis [51,52], regardless of the HCC etiology [50]. As to the PPP, the oxidative and non-oxidative branches of this pathway, controlled by G6PD, transaldolase (TALDO) and transketolase (TKT) enzymes, were elevated in human HCC [26,27]. Increased G6PD expression levels were reported in HCC patients in different studies [53,54] and correlated with grading, metastasis and poor prognosis, but not with etiology [54]. In addition, an enhanced expression of G6PD was found in HBV-associated HCC patients [55], and a comparison of the expression of the PPP enzymes in human HCC and the adjacent surrounding liver by transcriptome analysis supported previous findings showing their significant up-regulation in human HCCs [56]. On the other hand, lower G6PD expression was detected in patients who received sorafenib treatment after surgical removal of HCC and was significantly associated with better progression-free survival and overall survival [53].

As to the final step of glycolysis, which produces pyruvate and is catalyzed by pyruvate kinases (PKs), recent studies demonstrated that the expression of PKM2 was up-regulated in HCCs and correlated with a high TNM stage and level of vascular invasion [57,58]. The maintenance of high levels of glycolysis requires lactate excretion from the cells by a group of monocarboxylic acid transporters; accordingly, the expression levels of monocarboxylic acid transporter 4 (MCT4), which accelerates the export of lactate, were significantly higher in HCC tissues than in adjacent non-tumor tissues and positively correlated with tumor size poor overall survival (OS) and time to recurrence (TTR) [42]. 

Interestingly, the analysis of the biochemical events associated with the cirrhosis-the end-stage of chronic liver disease and the precancerous state [59,60] has been recently performed [61]. This study revealed that representative glycolytic enzymes, such as HK2, aldolase A (ALDOA) and PKM2 were significantly up-regulated in cirrhotic livers when compared with healthy liver, while also being associated with risk of HCC development. On the other hand, transcript levels of genes involved in OXPHOS remained unchanged in cirrhosis when compared to normal liver [61].

## 2. Animal Models of Hepatocarcinogenesis

The recent gene expression profiling of poorly-differentiated (HLE, HLF, SNU-449) and well-differentiated (Huh7, HepG2, Hep3B) HCC cell lines established that the cell lines, representative of well- and poorly-differentiated HCC subclasses, mimic, at least in part, the expression pattern of human HCC tissue-derived metabolic genes (HMGs) [62]. In particular, poorly differentiated cell lines more closely mimic human HCC profiles. 

Although a number of studies addressing the role of cancer metabolism and HCC metabolic reprogramming have been performed on cultured cancer cells, the use of cell lines cannot truly recapitulate the complexity of primary tumors, for several reasons: (1) culturing of tumor cells introduces genetic alterations not present in the original tumor; (2) HCC cells neither reproduce the complexity of the metabolic tumor microenvironment nor the tumor cell heterogeneity that is observed in vivo. In addition, hepatocarcinogenesis, similar to most human tumors, is a multistage process; therefore, investigation of fully transformed cells at their final step of progression does not allow to discriminate between changes that are the consequence of full cell transformation from those that are driving the tumorigenic process. Considering the fact that studies on early HCC stages in humans are hindered by the clinical difficulty of diagnosing preneoplastic lesions, animal models allowing to dissect the several steps of HCC are mandatory. 

Among the preclinical investigations, numerous studies using experimental animal models of hepatocarcinogenesis contributed to characterize the molecular mechanisms underlying HCC development and progression. A wide range of rodent animal models, that allow to study the impact of metabolic changes on HCC development are currently available [63]. Although no animal model can be considered as an “ideal” one for all HCC research purposes [64], several ones provide new insight concerning the metabolic profile of HCC. Such models include mainly: (i) chemically-induced models of hepatocarcinogenesis; (ii) genetically engineered mouse (GEM) models, with transgenic and knockout (KO) mice; (iii) xenograft mouse models; and (iv) patients derived xenografts (PDX). The particular advantage of some models is that they provide the opportunity to mimic and follow the complex multistep process of hepatocarcinogenesis. Clearly, no animal model closely recapitulates the multistep process of human hepatocarcinogenesis. Indeed, while GEM models are invaluable in studying specific molecular and signaling pathways interactions occurring in HCC development, their utility is limited by long incubation times and, often, by the lack of underlying liver disease, such as steatosis, fibrosis and cirrhosis. With a few exceptions [65], mouse models suffer the difficulty of enabling to dissect the several stages of human hepatocarcinogenesis, as HCCs often develop in the absence of evidence of preneoplastic lesions, such as those seen in humans (low-grade and high-grade dysplastic nodules). Thus, while human HCC occurs long after the appearance of initial preneoplastic nodules, mouse HCC develops suddenly and in the absence of clearly detectable progenitor lesions. On the other hand, although rat models do not allow genetic manipulations, they do offer some advantages compared to mouse models: (1) surgical procedures (i.e., bile duct ligation, partial hepatectomy) and, (2) non-invasive monitoring of HCC development are easier in rats than mice. In addition, rat models allow to follow the clonal expansion of initiated cells and their evolution to foci/nodules and then HCC, thanks to a great number of preneoplastic markers, such as gamma-glutamyl transpeptidase (GGT), glutathione S-transferase, placental form (GSTP), cytokeratin-19 (KRT-19), and glucose-6-phosphatase (G6Pase). This last aspect is highly relevant as it potentially allows to modulate the hepatocarcinogenesis process at well-defined times, therefore identifying effective anti-cancer treatments. 

**Figure 1:**
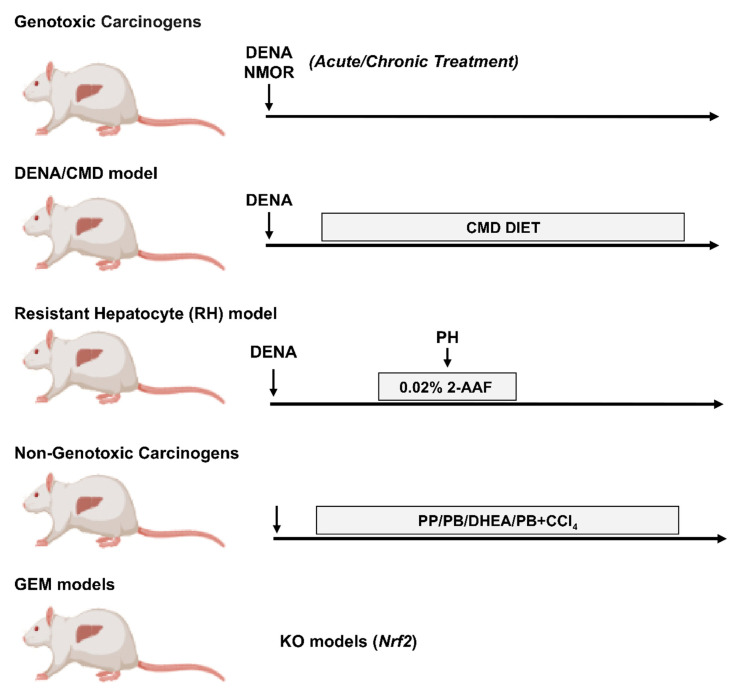
Examples of rat models used for the study of metabolic reprogramming in HCC. DENA: diethylnitrosamine; CMD: choline-devoid methionine-deficient diet; PH: partial hepatectomy; 2-AAF: 2-acetylaminofluorene; PP: Peroxisome Proliferator; PB: Phenobarbital; DHEA: dehydroepiandrosterone; CCl_4_: carbon tetrachloride; KO: knockout; Nrf2: nuclear factor, erythroid 2 like 2. The figure has been prepared by adapting BioRender images.

**Figure 2:**
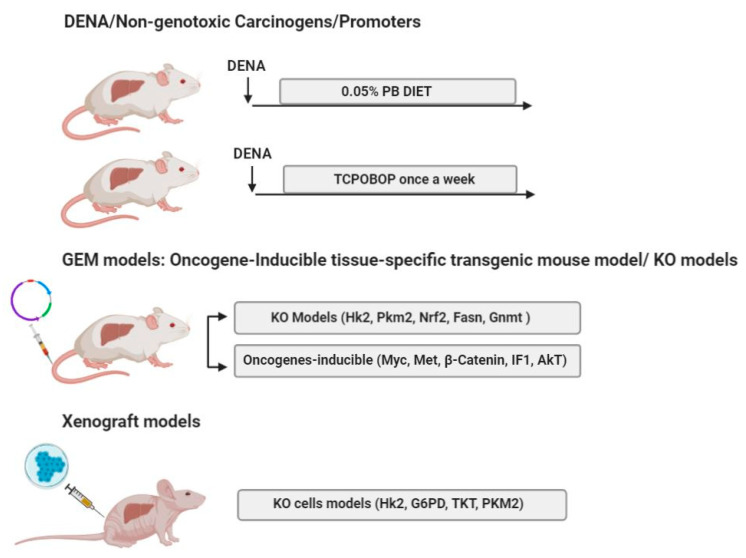
Examples of mouse models used for the study of metabolic reprogramming in HCC. DENA: diethylnitrosamine; PB: phenobarbital; TCPOBOP: 1,4-Bis[2-(3,5-dichloropyridyloxy)]benzene; Hk2: hexokinase 2; Pkm2: pyruvate kinase M2; Nrf2: nuclear factor, erythroid 2 like 2; Fasn: fatty acid synthase; Gnmt: glycine N-methyltransferase; IF1: ATPase Inhibitory Factor 1; AKT: protein-kinase B; G6pd: glucose-6-phosphate dehydrogenase; Tkt: transketolase. 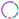 Hydrodynamic Injection. 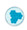 Cancer cells. The figure has been prepared by adapting BioRender images.

In the next sections, we will discuss various rat (Figure 1) and mouse (Figure 2) models, that among the overwhelming amount of available literature, may contribute to better define our knowledge on the metabolic alteration in HCC. 

## 3. Chemically-Induced Models of HCC

Historically, the metabolic changes, that captured initially the greatest attention in foci of altered hepatocytes induced by chemical carcinogens were those related to carbohydrate metabolism. In fact, one of the most significant early metabolic changes in rat hepatocarcinogenesis following exposure to the genotoxic hepatocarcinogen N-nitrosomorpholine (NMOR) was an excessive focal glycogen storage [66,67]. Of particular interest was that glycogen storage foci usually showed a reduction in the activities of G6Pase, while G6PD, the key enzyme of the PPP, was increased in its content and/or activity, thus indicating adaptive metabolic changes redirecting the altered carbohydrate metabolism towards other metabolic pathways, such as the PPP [66,68]. Increased G6PD levels have been also described in rat preneoplastic and neoplastic lesions generated by continuous administration of NMOR [69,70,71]. G6PD-positive preneoplastic lesions had also a significantly higher labelling index than that of G6PD-negative ones [69]. Interestingly, G6PD levels were also elevated in rat preneoplastic lesions induced by another carcinogen N-ethyl-N-hydroxyethylnitrosamine (EHEN), while the activities of the mitochondrial-bound enzyme, succinate dehydrogenase (SDH) resulted to be reduced [72].

## 4. Rat Models of HCC 

### 4.1. DENA

Among the several carcinogens used to induce liver cancer, such as N-methyl-N-nitrosourea (MNU), dimethylhydrazine (DMH), benzo(a)pyrene (BP) and others [73,74,75]. N-Nitrosodiethylamine (DENA) is the most widely used (Figure 1). Indeed, DENA is a carcinogen with the potential to produce tumors in every animal species tested and in various organs, including the liver, gastrointestinal and respiratory tract. DENA causes genomic mutations by forming ethyl adducts at many DNA positions with persistent and pro-mutagenic ethyl at thymine O4 and guanine O6 [76,77]. Although it has been demonstrated that DENA alone is sufficient to induce liver cancer in rodents, DENA-induced hepatocarcinogenesis varies with the age, sex, animal strain and the time of appearance of lesions [65,78]. Interestingly, gene expression patterns in DENA-induced mouse HCC result similar to those of the poorer survival group of human HCCs [79]. A more detailed description of DENA-induced models has been recently reviewed [64,80].

With regard to the general metabolic changes observed in DENA-induced models of hepatocarcinogenesis in rats, by examining gas chromatography/time-of-flight mass spectrometry (GC/TOFMS)-based metabolomics, serum and urine metabolites, Li et al. [81] performed a comparative analysis of normal control rats, HCCs and HCCs with lung metastasis (HLM), induced by chronic exposure to DENA. The authors demonstrated that glycolysis products and intermediates resulted elevated, while the TCA cycle was inhibited, in both HCC and HLM. Using the same experimental protocol, the metabolite profiles of extracts from tumor tissue, obtained through proton nuclear magnetic resonance (1H-NMR)-based metabolomics, showed an increased rate in glycolytic metabolism in HCC and metastasis vs. control livers [82]. Previous studies unveiled the implication of the PPP in DENA-induced rat preneoplastic lesions, as revealed by increased G6PD and TKT activity when compared with the surrounding tissue [83]. 

### 4.2. DENA/Choline-Deficient, Methionine-Restricted Diet (CMD) Model

Recently, PPP involvement has been reported in another rat model consisting of a single initiating dose of DENA followed by a choline-deficient, methionine-restricted (CMD) diet. [84], This nutritional regimen originally developed by Lombardi et al. [85] leads to a rapid accumulation of triglycerides (TGs) in hepatocytes and represents a frequently employed animal model presenting close pathological and biochemical similarities to human non-alcoholic steatohepatitis (NASH) [86]. In rats pre-treated with DENA, CMD diet effectively promotes the evolution of initiated cells to foci of altered GGT-positive hepatocytes and strongly enhances HCC development [87,88]. The study by Orrù et al. [84] revealed that preneoplastic lesions obtained with DENA+CMD model and identified by the marker GSTP, whose expression is undetectable in differentiated hepatocytes [89], demonstrated a significantly higher expression of G6PD. Moreover, co-expression of GSTP and G6PD in preneoplastic nodules demonstrated a higher proliferative capacity of these lesions. In this context, it is important to stress that an interesting study demonstrated that G6PD is directly activated by the transcription factor Nrf2 (NFE2L2, nuclear factor (erythroid-derived-2)-like 2 [90]. The same study revealed that the Keap1–Nrf2 (Kelch-like ECH-associated protein 1- Nrf2) system plays a central role in the protection of cells against oxidative and xenobiotic stresses [91]. Importantly, oxidative stress has emerged as a crucial player in HCC development and progression as 90% of HCC arise in the context of chronic liver inflammation, which leads to oxidative stress, generating excess reactive oxygen species (ROS) [92,93]. Nrf2-Keap1 system appeared also as an important metabolic regulator that redirects glucose and glutamine into the anabolic PPP in cancer cells [90]. Along these lines, several studies reported that NRF2 overexpression and/or the somatic mutations take place in many human cancers, including HCC, for a review see [94], and, remarkably, a very high frequency of Nrf2 somatic mutations associated with increased G6PD expression occurs at early stages of hepatocarcinogenesis in the DENA+CMD model [84,95]. 

### 4.3. DENA/Resistant Hepatocyte (RH) Model 

The most relevant observations regarding metabolic reprogramming in the process of rat hepatocarcinogenesis stem from studies employing the Resistant-Hepatocyte (RH) model [96]. The main advantage of the RH model is that it offers the possibility to identify distinct lesions (early preneoplastic foci, preneoplastic nodules, early and fully developed HCCs) at well-defined timings, representing an ideal preclinical tool for the study of molecular events occurring at very early stages of hepatocarcinogenesis. Due to the clinical difficulty of diagnosing early lesions in humans, the RH model undoubtedly helps to characterize the process of HCC development. In this experimental model, tumors are initiated by a single dose of DENA and promoted by a short-term dietary exposure to 2-acetylaminofluorene (2-AAF), that suppresses growth of all normal hepatocytes, coupled with 2/3 partial hepatectomy (PH) [96]. In these conditions, only DENA-initiated cells undergo proliferation, giving rise to early preneoplastic foci (EPF), small spherical lesions consisting of 15–100 hepatocytes. EPF can be identified by several preneoplastic markers, including GSTP, as early as 7 days after PH. Ten weeks from DENA initiation, a subset of GSTP-positive preneoplastic lesions expresses the putative progenitor/stem cell marker KRT-19 [97], which is considered a prognostic marker of poor outcome of HCC [98,99]. Comparative functional genomics showed co-clustering of KRT-19-positive early preneoplastic nodules and advanced rat HCCs with human HCCs characterized by poor prognosis. Furthermore, KRT-19-associated gene expression signature predicted patient survival and tumor recurrence [98,100], conferring a translational value to this model. Through the analysis of these KRT-19+ lesions, Kowalik et al. [54] demonstrated that not only a shift from OXPHOS to glycolysis represents a very early event in the hepatocarcinogenic process, but also, that PPP activation plays a role in the onset and progression of the process. Among the key molecules involved in the metabolic reprogramming of early preneoplastic lesions were G6PD and TRAP1, a mitochondrial molecular chaperone of the Hsp90 family that down-regulates both respiratory complex IV and succinate dehydrogenase (SDH) [101,102,103]. The authors further demonstrated that the consequent rise in intracellular succinate levels in preneoplastic nodules induces the stabilization of a key regulator of the glycolytic response, the transcription factor *hypoxia-inducible factor 1* (HIF-1α) [104], thus shifting the burden of ATP production to glycolysis [54]. These observations were paralleled by increased citrate synthase (CS) enzymatic activity, leading to an increase of citrate, an important allosteric inhibitor of phosphofructokinase 1 (PFK1) and SDH [105], and therefore inhibition of the late glycolytic steps and OXPHOS. With regard to the PPP, elevated G6PD levels were observed in rat early preneoplastic nodules and rat HCCs induced by the RH protocol [54]. Metabolic changes leading to increased G6PD expression were associated with a higher proliferative capacity of KRT-19+ preneoplastic lesions. Interestingly, induction of hepatocyte proliferation per se did not appear to be sufficient to boost PPP activation as shown by a decrease of G6PD mRNA and activity during liver regeneration occurring following 2/3 partial hepatectomy. Overall, these data demonstrate that a metabolic shift towards PPP induction and OXPHOS inhibition (1) is an early event in hepatocarcinogenesis, (2) is not required for normal hepatocyte proliferation, (3) is characteristic of the tumorigenic process and identifies specifically the most aggressive KRT-19+ preneoplastic lesions and not the KRT-19-negative ones. These results suggest the potential utility of the RH model to investigate the critical metabolic changes underlying HCC development.

### 4.4. Non-Genotoxic Compounds

Apart from genotoxic (direct-acting) carcinogens, such as DENA or NMOR [77], a variety of non-genotoxic carcinogens has been used in animal models of hepatocarcinogenesis. These compounds do not interact directly with DNA but are capable of inducing cancer by some secondary mechanism not related to direct gene damage [106,107]. Non-genotoxic carcinogens are believed to induce tumorigenesis by inducing ROS, causing cellular structures damage, increasing the risk of genetic error, affecting the proliferation, differentiation and apoptosis. These compounds include, for example, peroxisome proliferators (PPs), agonists of the constitutive androstane nuclear receptor (CAR), such as phenobarbital (PB) and 1,4-Bis[2 -(3,5-dichloropyridyloxy)]benzene (TCPOBOP), adrenal steroid hormones, such as dehydroepiandrosterone (DHEA), and drugs causing liver injury, such as thioacetamide (TAA), and carbon tetrachloride (CCl_4_) [106,107,108,109,110]. Interestingly, preneoplastic lesions and HCCs induced by PPs, as well as DHEA, unlike PB, are negative to marker enzymes, such as GSTP and GGT, classically associated to hepatocarcinogenesis induced by genotoxic carcinogens [111,112]. Nevertheless, the PPs and DHEA-induced preneoplastic lesions, similar to what seen with genotoxic carcinogens, are negative to G6Pase [111,112], the enzyme involved in gluconeogenesis and also in the synthesis of glucose, further supporting the notion that an increased glucose uptake appears to be a general metabolic feature in hepatocarcinogenesis. Oddly enough, in spite of increased glucose uptake, these preneoplastic lesions do not display increased PPP activation as demonstrated by their general negativity to G6PD staining [113], or inhibition of G6PD activity [114].

Studies aimed at investigating metabolic changes induced by non-genotoxic carcinogens in rat liver have been mainly addressed to modifications of lipid metabolism rather than to glucose consumption. Among the few examples is the work by Nishikawa et al. [115]. The authors used a rat model of cirrhosis induced by chronic administration of PB and CCl_4_, demonstrating that mitochondrial respiration was decreased in cirrhotic hepatocytes especially at late stages. In particular, hepatocytes isolated from rat liver with early signs of cirrhosis showed a metabolic shift from OXPHOS to glycolysis, while normal rat hepatocytes used OXPHOS for ATP generation [115].

## 5. Mouse Models of HCC

### 5.1. DENA/Non-Genotoxic Liver Tumor Carcinogens/Promoters

Experimental protocols employing DENA in combination with non-genotoxic compounds have been frequently used also with mice. Tumors obtained through such protocols are often characterized by a high frequency of β-catenin (*Ctnnb1*) mutations. A widely used model of mouse hepatocarcinogenesis is the DENA+PB protocol, which involves the selective clonal outgrowth of cells carrying β-catenin mutations with up to ~80% of tumors displaying mutation of this gene. Remarkably, DENA-induced liver tumors in the absence of PB treatment show only Ha-*ras* (~50%) or B-*raf* (20%)-mutations, with no β-catenin mutations [116]. Importantly, the Wnt/β-catenin signaling plays a relevant role in regulating glucose metabolism by inducing a shift from OXPHOS to glycolysis [117,118]. Using DENA+PB mouse experimental model, Unterberger et al. [119] demonstrated that both *Ha-ras* and *Ctnnb1*-mutated tumors showed a reduction in the levels of G6Pase, a condition supporting cancer cells since it utilizes glucose as s a source of energy. However, although glucose-6-phosphate may be utilized through the PPP, this resulted up-regulated in Ha-*ras*-, but not in *Ctnnb1*-mutated tumors. It is also puzzling that while transcriptional up-regulation of the TCA cycle enzymes, such as isocitrate dehydrogenase (IDH3a) and citrate synthase (CS) was observed in *Ctnnb1*-mutated tumors [119], increased lactate levels were observed in Ha-*ras* but not in *Ctnnb1*-mutated tumors. Similarly, Yuneva et al. did not detect [120] increased lactate levels in *MET*-induced mouse liver tumors, characterized by activating mutations of β-catenin [121]. Interestingly, we did not observe any changes in the levels of G6pd, the lactate transporter Mct4 and glucose transporter 1 (Glut1) were observed in HCCs generated in mice subjected to DENA+TCPOBOP protocol, despite the fact that ~85% of these HCCs carried β-catenin mutations [122]. It is of interest to note that even if the Wnt/β-catenin signaling has been reported to regulate energy metabolism [117], no difference in specific metabolic remodeling was observed in human G5 and G6 HCC subgroups, characterized by the Wnt/β-catenin pathway activation due to β-catenin (*CTNNB1*) mutation [37].

### 5.2. GEM Models, Oncogene-Inducible Tissue-Specific Transgenic Mouse Model

With the advent of new powerful technologies, genetically engineered animal models of HCC have been developed. However, besides few exceptions (i.e., p53 or Nrf2 KO rats) [123,124], genetically engineered animal models are limited to the mouse, and only few of these models have been employed to elucidate the role of metabolic reprogramming in liver cancer. Among the mouse protocols of hepatocarcinogenesis, *MYC*, *MET*, *β-catenin* are considered as canonical oncogenes whose increased expression, amplification, or pathway activation have been observed in several human cancers. In the context of this review, more relevant is that the increased expression of these oncogenes drives important metabolic changes. While *MYC* controls the expression of glycolytic enzymes, the Krebs cycle, mitochondrial respiration and glutaminase levels [125,126], the tyrosine kinase receptor MET deeply affects carbohydrate metabolism [127]. Among the key player of the Wnt pathway [128,129], β-catenin controls ammonia and glucose metabolism [117]. However, as already mentioned, that tumors harboring β-catenin mutation undergo metabolic reprogramming is questionable in view of the findings demonstrating that neither an increase of PPP, nor enhanced lactate levels were observed in tumors of mice subjected to DENA+PB or in *MET*-induced mouse liver tumors, characterized by activating mutations of β-catenin [119,120].

Using mouse models of liver cancer induced by tissue-specific overexpression of *MYC* [130] and *MET* [131], the effect of these two oncogenes on metabolic alterations in developed HCC were investigated by Yuneva and collaborators [120]. While *MYC*-induced mouse liver tumors significantly increased both glucose and glutamine catabolism, *MET*-induced liver tumors used glucose to produce glutamine. HK2, lactate levels. Moreover, the Krebs cycle intermediates (such as fumarate, malate and citrate) were significantly increased in liver tumors induced by *MYC* but not in those induced by *MET* [120]. However, it should be underlined that while *MYC*-induced mouse tumors resemble immature hepatoblastomas [130], tumors induced by *MET* display features typical of differentiated HCC [131]. Taken together, these results suggest that glucose and glutamine metabolism in liver tumors depends on the nature of the oncogene and confirmed heterogeneous behavior of the glutamine pathway in HCC [132]. Using a mouse model of *MYC*-induced liver cancer [130], it has been also observed that another important metabolic alteration in HCC is represented by a significant decrease in fatty acid oxidation (FAO) and an increase in the activity of pyruvate dehydrogenase (PDH), an enzyme complex that catalyzes the conversion of pyruvate into acetyl-CoA [133,134].

More recently, two transgenic mouse models contributed to deepen our knowledge regarding the implication of Nrf2-Keap1 pathway in metabolic reprogramming; mice expressing a KEAP1-resistant form of NRF2 in their hepatocytes (Nrf2^Act-hep^) and allowing hepatocyte-specific activation of NRF2, as well as mice that selectively overexpress p62 in hepatocytes, which activates Nrf2 [135]. In particular, p62-mediated NRF2 activation induced hepatic expression of numerous metabolic enzymes, involved in glycogen, glucose and PPP metabolism and de novo lipogenesis, such as G6PD, HK2, glucokinase (GCK), TALDO1, TKT, fatty acid synthase (FASN) [135]. Notably, these changes were impressively similar to those previously found in the most aggressive KRT-19+ preneoplastic rat hepatic lesions [54] further supports the notion that a metabolic switch towards glucose metabolism is associated to tumor progression.

Transgenic mouse models also allowed to investigate the impact of impaired OXPHOS on hepatocarcinogenesis, a question that has been rarely addressed in the literature. To this aim, Santacatterina et al. [136] subjected transgenic mice carrying the mutant active form of the ATPase Inhibitory Factor 1 (IF1), an inhibitor of the mitochondrial H^+^-ATP synthase [137] to the carcinogen DENA. Interestingly, the transgene expression caused OXPHOS inhibition by restraining both Complex IV and ATP synthase activity. Such inhibition resulted in a higher susceptibility to DENA-induced hepatocarcinogenesis. The increased tumor burden in IF1 transgenic mice was accompanied by increased proliferation and diminished apoptosis [136].

Genetically engineered mouse model of hepatocarcinogenesis, in which HA-tagged myristylated Akt (Myr-Akt) is stably expressed into the hepatocytes of wild-type mice, was also generated. In this model Calvisi et al. [138] demonstrated the simultaneous activation of AKT, mTOR, and ribosomal protein s6 (RPS6) along with lipogenic enzymes involved in fatty acid biosynthesis and cholesterol biosynthesis, thus highlighting the involvement of AKT–mTOR signaling pathway in the lipogenic process. At the same time, the upregulation of transcription factors promoting lipogenesis, such as carbohydrate-responsive element-binding protein (chREBP), Liver X receptor beta (LXR-β), sterol regulatory element-binding protein 1 (SREBP1) and Sterol regulatory element-binding protein 2 (SREBP2) was also established. Moreover, the overexpression of SREBP1 and SREBP2 correlated with the inactivation/phosphorylation of Glycogen Synthase Kinase 3 Beta (GSK-3β) and other anti-lipogenic signals. These results underline the involvement of AKT in the promotion of the lipogenic process.

### 5.3. Knockout (KO) Models

GEM technology disposes of knockout models, which are utilized for loss-of-function studies or conditional GEM, that involve the use of site-specific recombinase systems allowing to control the gene expression [64]. These models are particularly helpful if one wants to investigate the role of specific genes involved in metabolic reprogramming.

HK2: Although not all the molecular mechanisms leading to significant up-regulation of aerobic glycolysis are yet understood, hexokinases are recognized as the important players regulating glycolytic flux [139]. In fact, relevant observation was obtained following HK2 depletion. Similar to what observed with HK2 conditional knockout, in mouse models of K-Ras-driven non-small cell lung cancer (NSCLC) and ErbB2/Neu-driven breast cancer [140], genetic ablation of HK2 in liver-specific HK2 knockout (KO) mice (HK2 ^f/f^; *AlbCre* mice) decreased the incidence of tumors in a model of DENA-induced hepatocarcinogenesis. Reduction in tumor number was associated with a reduced rate of proliferation [141]. Mechanistically, HK2 ablation resulted in a marked inhibition of glucose flux, while glutamine flux and the TCA cycle were maintained. Noteworthy, the loss of HK2 led to a compensatory upregulation of OXPHOS [141]. The finding that enhanced OXPHOS was not able to sustain tumor growth supports the relevance of increased glucose uptake in HCC onset and progression.

PKM2: KO models have been also largely applied in order to study the requirement of PKM2 for HCC growth. Often, however, they yielded contradictory results. While aged germline PKM2-null mice (PKM2^−/−^) displayed a dramatic incidence of spontaneous HCC that was accompanied by altered systemic glucose homeostasis, inflammation, and hepatic steatosis [142], depletion of PKM2 in mice did not negatively affect c-*MYC*-induced tumorigenesis. These results suggested that increased PKM2 is not required to support c-*MYC*-induced hepatocarcinogenesis and probably different pyruvate kinase isoforms sustain liver cancer progression [143].

NRF2: Several important observations stemmed also from the Nrf2 KO mice and rats. Ngo et al. [144] showed that Nrf2 deficiency inhibited HCC development in DENA-treated Nrf2 KO mice. Nrf2 genetic disruption reduced the expression of PPP enzymes, such as *G6pd*, *Taldo1* and *Tkt*. The authors concluded that Nrf2 activation enhances the expression of genes involved in the uptake and redistribution of glucose into the PPP to support rapid cancer cell growth and proliferation. In addition, genetic inactivation of Nrf2 in rats [124] fully prevented the formation of preneoplastic foci in rats treated with DENA and CMD diet and was associated with the absent/low expression of Nrf2 target genes, such as *Gstp1* and *G6pd* [84]. The fact that Nrf2 plays a crucial role in HCC development by inducing metabolic reprogramming was recently supported by the finding that inhibition of the Keap1-Nrf2 pathway by thyroid hormone (T3) is associated with a switch from Warburg metabolism to OXPHOS which precedes regression of rat HCCs [145] (for a detailed description of the effect of T3, see chapter 7 entitled Pharmacological targeting of Warburg metabolism in HCC). The discovery of specific Nrf2 inhibitors, unfortunately lacking at present, will help to better elucidate the role of this transcription factor in HCC development and, hopefully, to efficiently impair the Nrf2-dependent metabolic reprogramming of neoplastic hepatocytes. The need for such studies becomes extremely urgent also on the basis of a very recent study [146] showing that the loss of protein kinase Cλ/ι (PKCλ/ι) promotes HCC by enhancing OXPHOS, in spite of activation of Nrf2—which, according to the literature, is known to redirect metabolism towards glycolysis [146].

FASN: Cancer cells not only rely on glucose but also depend on other metabolites such as glutamine, serine or fatty acids [147,148,149]. De novo lipogenesis together with an increased expression of fatty acid synthase (FASN), the crucial metabolic enzyme in fatty acid biosynthesis [150], represents another important alteration of the metabolic rewiring in HCC [151]. Interesting observations with KO models stemmed from the studies in which conditional FASN KO mice were used [150]. Through different oncogene-induced HCC mouse models, such as AKT, AKT/c-Met and c-Met/β-catenin models, it has been observed that genetic ablation of FASN totally abolished hepatocarcinogenesis driven by AKT and AKT/c-Met [150,152,153]. On the contrary, FASN was not required in c-Met/β-catenin-driven liver cancer development, suggesting that tumors might be either addicted to or independent from FASN activity depending on the nature of oncogenes used [150].

GNMT: With regard to amino acid metabolism, it has been reported that many pediatric patients showing liver disease also display mutations of Glycine N-methyltransferase (GNMT), an enzyme which catalyzes the excess of hepatic S-adenosylmethionine (SAMe), highlighting its implication in liver function. Martinez-Chantar et al. [154] found that GNMT-KO in mice leads to fatty liver, fibrosis and HCC concomitantly with elevated aminotransferase, methionine, and SAMe serum levels. They also demonstrated the correlation of GNMT with the Ras and Janus kinase (JAK)/signal transducer and activator of transcription (STAT) pathway since activation of this pathway increased in liver tumors arising from GNMT-KO mice together with the suppression of the Ras inhibitors Ras-association domain family/tumor suppressor (RASSF) 1 and 4 and the JAK/STAT inhibitors suppressor of cytokine signaling (SOCS) 1–3 and cytokine-inducible SH2-protein.

### 5.4. Xenograft Mouse Models

Xenograft mouse models are established by the implantation of either human tumor fragments or cultured cancer cells of human or other species, under the skin (ectopic) or into the organ of tumor origin (orthotopic) [64,155,156]. Subcutaneous xenograft models, obtained by subcutaneous injection of cancer cells into immunocompromised mice, are the most widely used mouse models in current HCC studies (for a detailed description, see [156]) This model, characterized by being easy to perform, highly reproducible, not expensive and with low procedure mortality [64], represents an important tool for a rapid in vivo screening of tumor growth and drug response, although considerable attention has to be taken into consideration while translating obtained results into the clinical practice [157].

With regard to the initial steps of glycolysis, using a Huh7 subcutaneous tumorigenesis assay, DeWaal et al. [141] demonstrated that mice inoculated with the HK2 shRNA cells and fed the doxycycline (Dox)-infused diet had significantly smaller tumors (about 50%) than those of the control groups. Several studies using xenograft mouse models have been performed to study the role of the PPP pathway in HCC development. Using both Huh7 mouse subcutaneous xenograft model and orthotopic model, which even better reproduces the tumor microenvironment and organ tropism [155,156], Hong et al. [53] demonstrated that G6PD suppression inhibited tumor growth, reinforcing the relevance of G6PD in hepatocarcinogenesis. To further investigate the impact of PPP on cancer growth in vivo, subcutaneous and orthotopic tumor models with TKT KO cells were also exploited [56]. While both models significantly suppressed the tumor growth, the orthotopic one also reduced the growth of metastatic lesions in the lungs [56]. Xenograft tumor models also helped to elucidate the role of PKM2 in liver tumorigenesis. Replacement of PKM2 with PKM1 isoform caused the reversal of the Warburg effect and reduced ability to form tumors in nude mouse xenografts [29]. Moreover, in vivo delivery of siRNAs, specific to PKM2, in SCID mice, led to a substantial reduction of tumor volume in established HepG2 xenografts [158].

**Figure 3:**
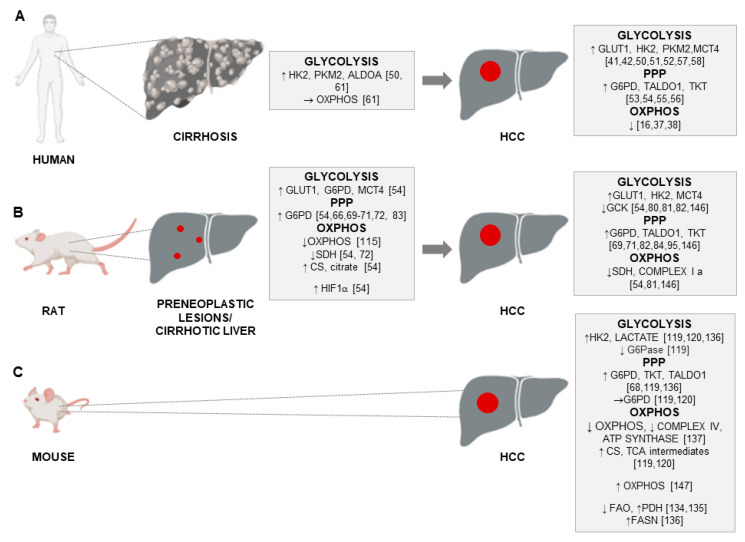
Principal metabolic changes observed in (**A**) human cirrhosis and HCC, (**B**) rat cirrhotic liver/preneoplastic lesions, rat HCC, as well as (**C**) in mouse HCC. The figure has been prepared by adapting BioRender images.

**Figure 4:**
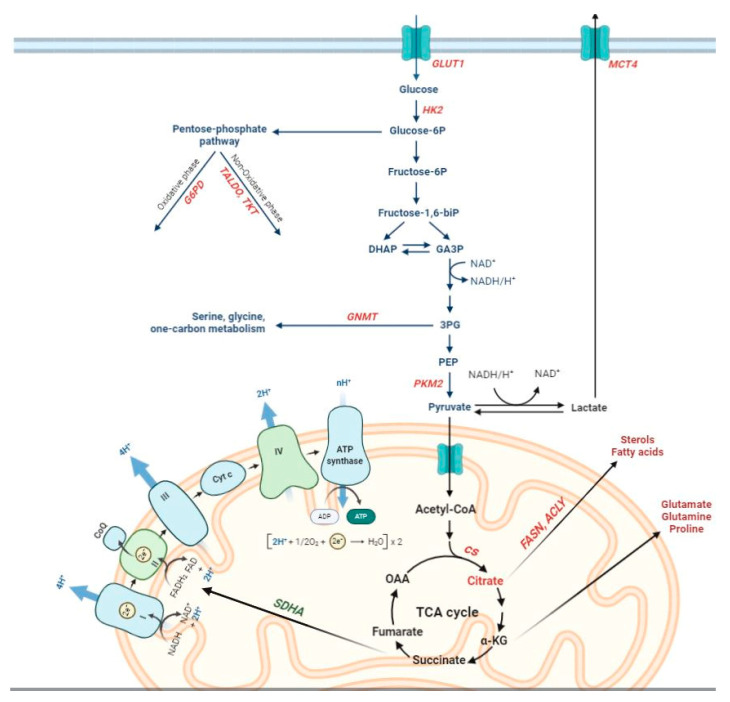
Schematic representation of altered biochemical pathways in rat preneoplastic nodules and HCC (red: up-regulation, green: down-regulation). The figure has been prepared by adapting BioRender images.

A summary of main metabolic changes reported in human and rat cirrhosis, rat preneoplastic lesions, human/rat/mouse HCC is reported in Figure 3. Moreover, altered biochemical pathways in rat preneoplastic nodules and HCC are depicted in Figure 4.

## 6. MicroRNA and Metabolic Reprogramming

Xenograft models have also helped to elucidate the role of miRNAs in metabolic reprogramming. Indeed, mounting evidence indicates that also microRNAs (miRNAs), whose aberrant expression represents a hallmark of human HCC, could regulate metabolic reprogramming observed in cancer cells [159].

In fact, miRNAs are capable to directly control the expression of glucose transporters (GLUT family), metabolic enzyme (HK2, aldolase A), and protein kinases (AMPK, PI3K), as well as indirectly regulate the expression of several transcriptional factors (p53, c-Myc) [159]. Importantly, some miRNAs have been confirmed in rodent models and tested as therapeutic targets [157]. With regard to HK2, Guo et al. [160] demonstrated that miRNA-199a-5p, down-regulated in human HCCs and regulated by HIF-1α, directly targets the 3′-untranslated region of HK2 and suppresses glucose consumption, lactate production, cell proliferation, as well as tumorigenesis. Indeed, a reduced volume and weight of the xenograft tumors was observed after inoculation of Huh7 cells overexpressing miR-199a-5p [160]. Recently, another study reported miR-885-5p, strongly downregulated in HCC, to regulate HK2 expression [161]. Forced expression of miR-885-5p not only significantly reduced the volume of the xenograft tumors but inhibited the expression of transcription factors, transporters and enzymes related to the Warburg metabolism, such as HIF-1a, GLUT1, HK2 and lactate dehydrogenase A (LDHA), a key enzyme involved in the conversion of pyruvate to lactate [161]. These results undoubtedly shed light on the possible application of miRNA/HK2 axis in HCC treatment.

Similarly, Zhao et al. [162] established that miR-145 regulates the expression of the lactate transporter MCT4, causing the accumulation of lactate within tumor cells in HCC. The main function of MCT4 is to promote the glycolysis-induced lactate stress through the transport into the extracellular environment of intracellular lactate. In this study, Zhao et al. [162] clarified the negative correlation between miR-145 and MCT4 expression levels in human HCC, thereby suggesting the potential role of miR-145 as a therapeutic target. They demonstrated that the expression of MCT4 was significantly reduced by the treatment of HepG2, Hep3B and HuH7 cells, with miR-145, therefore impeding the effusion of intracellular lactate and retarding the acidification of extracellular PH.

## 7. Pharmacological Targeting of Warburg Metabolism in HCC

Considering the abovementioned results, a possible approach in developing antitumoral therapies is to target either pathways activated by driver mutations or pathways that enable some tumor phenotypes, such as cancer metabolism [19,163]. Even if Warburg’s initial observations did not result in effective treatments for cancer, his studies had a deep impact on cancer research area [164]. In fact, from a therapeutic perspective, accumulating evidence of crosstalk between metabolic regulation and signaling pathways indicates that a combined inhibition of cancer metabolism and signaling pathways might be a powerful therapeutic option in HCC [165,166]. Several animal studies, reported below, demonstrate the advantage of targeting cancer metabolism on HCC development and progression.

Among the possible targets, there are those involved in the regulation of glycolysis. HK2 represents one of such targets. Focusing on a HK isoform that is expressed only by HCC cells and not by the normal hepatocytes could be used to selectively target HCC cells without altering metabolic functions in normal hepatocytes [49,140]. Several lines of evidence highlight and confirm the importance of targeting HK2 as a feasible approach for HCC treatment. In a study of C3H/He mice implanted with MH134 mouse HCC cells and successively treated with 3-BrPA, 3-bromopyruvate (3-BrPA), a HK2 inhibitor [167], mean tumor volume was shown to be significantly reduced [168]. Such anti-tumoral effect was associated with induction of apoptosis by 3-BrPA. Another proposed approach was based on HK2 depletion. Considering the fact that HK2 ablation in the liver led not only to a reduction in glycolysis but also resulted in up-regulation of OXPHOS, DeWaal et al. [141] proposed targeting of both metabolic pathways as potentially efficient therapeutic option for HCC. In fact, HK2 silencing and the treatment with complex I inhibitor, metformin [169], had a synergistic effect on subcutaneous tumor growth in vivo [141]. Furthermore, the authors observed that, the use of FDA-approved therapeutic drug for HCC, sorafenib coupled with HK2 depletion decreased tumor growth more extensively that each standalone treatment. when compared to each treatment alone. HK2 depletion also sensitized HCC cells to cell death, suggesting that it can increase the efficacy of sorafenib treatment [141]. Sensitization of HCC cells to sorafenib treatment has been also reported following genetic knockdown and pharmacological inhibition of TKT by a thiamine antagonist, oxythiamine (OT), in subcutaneous xenografts [56].

In a recent study [170], FAO was shown to represent a possible druggable metabolic pathway for HCC treatment. A brief exposure to medium-chain or long-chain high-fat diets improved the survival of mice with c-Myc-driven HCC, characterized by a significant decline in FAO and an increase of PDH levels. Short exposure to both diets not only normalized FAO and PDH activities, but also influenced the expression of more than 600 tumor-dysregulated transcripts, mainly involved in the control of cell cycle, proliferation and metabolism. Once again, so-called ‘normalization of the Warburg effect’ was accompanied by a significant down-regulation of 6-phosphogluconate dehydrogenase (6PGD), the second enzyme of the oxidative brunch of PPP, and reduced expression of Glut1 [170].

Considering the relevance of Wnt signaling in hepatocarcinogenesis and the role of β-catenin in the regulation of genes of glutamine metabolism [171], Adebayo Michael et al. [172] proposed a novel therapeutic approach to unsettle tumor metabolism and contrast β-catenin-mutated liver tumors. These tumors exhibit positivity for glutamine synthetase (GS) and p-mTOR-S2448, an indicator of mammalian target of rapamycin complex 1 (mTORC1) activation. Using Met-β-catenin mouse model, in which mice harbor mutant β-catenin and display c-Met co-expression by sleeping beauty transposon/transposase and hydrodynamic tail vein injection (SB-HTVI), a significant decrease in tumor volume was observed in the group treated with rapamycin, an inhibitor of mTORC1 [173]. Successfully, the authors treated the mice with both rapamycin and GC-1, a thyroid hormone receptor beta-specific agonist [174] with partial Met-inhibitory activity [175]. A significant reduction of tumor burden when compared to the control group was observed, indicating that simultaneous targeting of β-catenin-GS-mTORC1 axis in liver tumors may be a valid treatment option [172].

The importance of rewiring of cellular metabolism from enhanced glycolytic phenotype towards OXPHOS has been recently reported by Kowalik et al. [145]. Encouraging results have been observed in the RH model following treatment with thyroid hormone 3,5,3′-triiodo-L-thyronine (T3). Thyroid hormones (THs), 3,5,3′,5′-tetraiodo-L-thyronine (thyroxine or T4) and T3, are known to influence a multiplicity of physiological processes, including development, cell growth and proliferation. The profound impact of THs on cellular metabolic processes and energetic homeostasis in almost all tissues has been widely recognized, too [176,177]. Moreover, proteomics and transcriptomic studies reported that approximately 8% of total liver proteins are regulated by THs in vivo [178]. Most of the THs effects are mediated by the thyroid hormone nuclear receptors (TRs) TRα and TRβ and the latter one represents the most abundant isoform in the liver [179]. It has been observed that a 7-day treatment with T3 of rats bearing hepatic preneoplastic nodules characterized by local hypothyroidism [180] and Warburg phenotype [54], induced their rapid regression [181]. T3 administration severely affected the expression of metabolic genes, and particularly, those involved in the PPP and mitochondrial respiration, as early as two days after treatment [145]. Even more striking was the fact that T3 administration to HCC bearing rats induced tumor regression by prompting a metabolic reprogramming [145]. Feeding T3-supplemented diet resulted in a clear switch from high Hk2 expression to that of glucokinase (Gck), accompanied by Glut1, Mct4, PPP enzymes down-regulation and increased activity of complex I and II of the respiratory chain. Moreover, T3 treatment greatly reduced tumor growth in a xenograft mouse model. This finding seems particularly important from a translational point of view as HCC is a tumor type that is often diagnosed at late and advanced stages when it is no longer amenable to curative approaches. These results are in line with previous studies demonstrating antitumoral effect of T3 in other experimental animal models [182,183], as well with those indicating a profound impact of T3 on mitochondrial function by regulating processes such as mitogenesis, proton leak and increasing OXPHOS [184,185]. For the abovementioned reasons, T3 or thyromimetics (such as GC-1), devoid of the cardiac toxic effects of thyroid hormone [186], would be worth of testing in clinical trials as they might improve the prognosis of patients with HCC.

Although at present the inhibition of the PPP is hampered by the lack of specific inhibitors and their limited clinical application, several studies showed that the uncompetitive G6PD inhibitor, dehydroepiandrosterone (DHEA) [187] inhibited the growth of early pre-neoplastic liver lesions, delayed the progression to HCC of persistent liver nodules induced by the RH model and was associated with a marked decrease of liver G6PD activity [188,189]. Interestingly, a small molecule (G6PDi-1) that effectively inhibits G6PD was identified very recently [190]. Hopefully, the discovery of such specific G6PD inhibitor will allow to definitely establish the role of the oxidative branch of PPP in HCC onset and progression.

## 8. Conclusions

Metabolic reprogramming, characterized by a striking surge in the rate of glycolysis and PPP concomitantly with decreased OXPHOS, appears to be a critical event in the pathogenesis of HCC, the main type of primary liver cancer. Despite these observations, the role of metabolic changes in HCC onset and progression has received less attention than it deserves. Thus, key questions on the significance of metabolic alterations in cancer remain unsolved. Critically, most of the studies have been performed either on tumor cell lines or on xenografts of cancer cell lines and fully developed cancers, therefore whether metabolic rewiring occurs in the early stages of neoplastic progression driving the tumorigenic process or are just bystander effects of deregulated oncogenic signaling pathways remains uncertain. Thus, although no rodent model can fully recapitulate the process of human hepatocarcinogenesis, they can prove to be particularly useful as they offer the possibility to dissect the several steps of hepatocarcinogenesis. In this context, it is important to note that the gene expression signature of mice exposed to chronic treatment with DENA resembled the signature of human HCCs characterized by poor prognosis [79]. In addition, early preneoplastic nodules generated through the RH rat model displayed the same miRNomic and transcriptomic profile of human HCC characterized by the worse prognosis [100,191].

Animal models are also important as they are able to challenge tenets stemming from the literature. As an example, in spite of a large body of evidence indicating that metabolic reprogramming from OXPHOS to Warburg phenotype is a common feature of HCC, very recent studies with KO mice implicate that enhanced OXPHOS may play a key role in HCC progression [146]. The fact that enhanced OXPHOS occurs concomitantly with Nrf2 activation–a transcription factor that is known to redirect oxidative metabolism towards a Warburg phenotype–represents a relevant exception to the general concept of metabolic reprogramming and poses the question as to whether increased glucose uptake and activation of PPP can be induced concomitantly with enhanced mitochondrial respiration.

In conclusion, animal models represent a powerful tool for improving our understanding of the role and the relevance of metabolic changes in HCC onset and progression. By using different species and by combining different models it is possible to extrapolate information that are difficult to unveil in humans. In addition, it is possible to address the fundamental question as to whether metabolic changes are simply a consequence of mutations of oncogenes/tumor suppressor genes or are, by themselves, key events in HCC development. Moreover, the evidence provided in this review indicates the possible translational relevance of metabolic alterations for HCC treatment stemming from different rodent models of HCC.

## References

[1] Bray F., Ferlay J., Soerjomataram I., Siegel R.L., Torre L.A., Jemal A. (2018). Global cancer statistics 2018: GLOBOCAN estimates of incidence and mortality worldwide for 36 cancers in 185 countries. CA Cancer J. Clin..

[2] El-Serag H.B., Rudolph K.L. (2007). Hepatocellular carcinoma: Epidemiology and molecular carcinogenesis. Gastroenterology.

[3] Llovet J.M., Ricci S., Mazzaferro V., Hilgard P., Gane E., Blanc J.F., de Oliveira A.C., Santoro A., Raoul J.L., Forner A. (2008). SHARP Investigators Study Group. Sorafenib in advanced hepatocellular carcinoma. N. Engl. J. Med..

[4] Finn R.S., Merle P., Granito A., Huang Y.H., Bodoky G., Pracht M., Yokosuka O., Rosmorduc O., Gerolami R., Caparello C. (2018). Outcomes of sequential treatment with sorafenib followed by regorafenib for HCC: Additional analyses from the phase III RESORCE trial. J. Hepatol..

[5] Llovet J.M., Montal R., Sia D., Finn R.S. (2018). Molecular therapies and precision medicine for hepatocellular carcinoma. Nat. Rev. Clin. Oncol..

[6] Huang A., Yang X.R., Chung W.Y., Dennison A.R., Zhou J. (2020). Targeted therapy for hepatocellular carcinoma. Signal. Transduct. Target. Ther..

[7] Gao Q., Huang X.Y., Qiu S.J., Yamamoto I., Sho M., Nakajima Y., Zhou J., Li B.Z., Shi Y.H., Xiao Y.S. (2009). Overexpression of PDL-1 significantly associates with tumor aggressiveness and post-operative recurrence in human hepatocellular carcinoma. Clin. Cancer Res..

[8] Calderaro J., Rousseau B., Amaddeo G., Mercey M., Charpy C., Costentin C., Luciani A., Zafrani E.S., Laurent A., Azoulay D. (2016). Programmed death ligand 1 expression in hepatocellular carcinoma: Relationship with clinical and pathological features. Hepatology.

[9] Yarchoan M., Xing D., Luan L., Xu H., Sharma R.B., Popovic A., Pawlik T.M., Kim A.K., Zhu Q., Jaffee E.M. (2017). Characterization of the Immune Microenvironment in Hepatocellular Carcinoma. Clin. Cancer Res..

[10] El-Khoueiry A.B., Sangro B., Yau T., Crocenzi T.S., Kudo M., Hsu C., Kim T.Y., Choo S.P., Trojan J., Welling T.H. (2017). Nivolumab in patients with advanced hepatocellular carcinoma (CheckMate 040): An open-label, non-comparative, phase 1/2 dose escalation and expansion trial. Lancet.

[11] Nault J.C., Mallet M., Pilati C., Calderaro J., Bioulac-Sage P., Laurent C., Laurent A., Cherqui D., Balabaud C., Zucman-Rossi J. (2013). High frequency of telomerase reverse-transcriptase promoter somatic mutations in hepatocellular carcinoma and preneoplastic lesions. Nat. Commun..

[12] Boyault S., Rickman D.S., de Reyniès A., Balabaud C., Rebouissou S., Jeannot E., Hérault A., Saric J., Belghiti J., Franco D. (2007). Transcriptome classification of HCC is related to gene alterations and to new therapeutic targets. Hepatology.

[13] Guichard C., Amaddeo G., Imbeaud S., Ladeiro Y., Pelletier L., Maad I.B., Calderaro J., Bioulac-Sage P., Letexier M., Degos F. (2012). Integrated analysis of somatic mutations and focal copy-number changes identifies key genes and pathways in hepatocellular carcinoma. Nat. Genet..

[14] Schulze K., Imbeaud S., Letouzé E., Alexandrov L.B., Calderaro J., Rebouissou S., Couchy G., Meiller C., Shinde J., Soysouvanh F. (2015). Exome sequencing of hepatocellular carcinomas identifies new mutational signatures and potential therapeutic targets. Nat. Genet..

[15] Zucman-Rossi J., Villanueva A., Nault J.C., Llovet J.M. (2015). Genetic Landscape and Biomarkers of Hepatocellular Carcinoma. Gastroenterology.

[16] Cancer Genome Atlas Research Network (2017). Electronic address: Wheeler@bcm.edu; Cancer Genome Atlas Research Network. Comprehensive and Integrative Genomic Characterization of Hepatocellular Carcinoma. Cell.

[17] Midorikawa Y., Yamamoto S., Tatsuno K., Renard-Guillet C., Tsuji S., Hayashi A., Ueda H., Fukuda S., Fujita T., Katoh H. (2020). Accumulation of molecular aberrations distinctive to hepatocellular carcinoma progression. Cancer Res..

[18] Cairns R.A., Harris I.S., Mak T.W. (2011). Regulation of cancer cell metabolism. Nat. Rev. Cancer.

[19] Mayers J.R., Vander Heiden M.G. (2017). Nature and Nurture: What Determines Tumor Metabolic Phenotypes?. Cancer Res..

[20] Warburg O., Wind F., Negelein E. (1927). The metabolism of tumors in the body. J. Gen. Physiol..

[21] Warburg O. (1956). On the origin of cancer cells. Science.

[22] Hanahan D., Weinberg R.A. (2011). Hallmarks of cancer: The next generation. Cell.

[23] Hsu P.P., Sabatini D.M. (2008). Cancer cell metabolism: Warburg and beyond. Cell.

[24] Schulze A., Harris A.L. (2012). How cancer metabolism is tuned for proliferation and vulnerable to disruption. Nature.

[25] Vander Heiden M.G., Cantley L.C., Thompson C.B. (2009). Understanding the Warburg effect: The metabolic requirements of cell proliferation. Science.

[26] Patra K.C., Hay N. (2014). The pentose phosphate pathway and cancer. Trends Biochem. Sci..

[27] Kowalik M.A., Columbano A., Perra A. (2017). Emerging Role of the Pentose Phosphate Pathway in Hepatocellular Carcinoma. Front. Oncol..

[28] Mazurek S., Boschek C.B., Hugo F., Eigenbrodt E. (2005). Pyruvate kinase type M2 and its role in tumor growth and spreading. Semin. Cancer Biol..

[29] Christofk H.R., Vander Heiden M.G., Harris M.H., Ramanathan A., Gerszten R.E., Wei R., Fleming M.D., Schreiber S.L., Cantley L.C. (2008). The M2 splice isoform of pyruvate kinase is important for cancer metabolism and tumour growth. Nature.

[30] Sullivan L.B., Gui D.Y., Vander Heiden M.G. (2016). Altered metabolite levels in cancer: Implications for tumour biology and cancer therapy. Nat. Rev. Cancer..

[31] Mazurek S. (2011). Pyruvate kinase type M2: A key regulator of the metabolic budget system in tumor cells. Int. J. Biochem. Cell Biol..

[32] Anastasiou D., Poulogiannis G., Asara J.M., Boxer M.B., Jiang J.K., Shen M., Bellinger G., Sasaki A.T., Locasale J.W., Auld D.S. (2011). Inhibition of pyruvate kinase M2 by reactive oxygen species contributes to cellular antioxidant responses. Science.

[33] Shang R.Z., Qu S.B., Wang D.S. (2016). Reprogramming of glucose metabolism in hepatocellular carcinoma: Progress and prospects. World J. Gastroenterol..

[34] Nicholson J.K., Lindon J.C., Holmes E. (1999). ‘Metabonomics’: Understanding the metabolic responses of living systems to pathophysiological stimuli via multivariate statistical analysis of biological NMR spectroscopic data. Xenobiotica.

[35] Griffin J.L. (2006). Understanding mouse models of disease through metabolomics. Curr. Opin. Chem. Biol..

[36] Yang Y., Li C., Nie X., Feng X., Chen W., Yue Y., Tang H., Deng F. (2007). Metabonomic studies of human hepatocellular carcinoma using high-resolution magic-angle spinning 1H NMR spectroscopy in conjunction with multivariate data analysis. J. Proteome Res..

[37] Beyoğlu D., Imbeaud S., Maurhofer O., Bioulac-Sage P., Zucman-Rossi J., Dufour J.F., Idle J.R. (2013). Tissue metabolomics of hepatocellular carcinoma: Tumor energy metabolism and the role of transcriptomic classification. Hepatology.

[38] Bayard Q., Meunier L., Peneau C., Renault V., Shinde J., Nault J.C., Mami I., Couchy G., Amaddeo G., Tubacher E. (2018). Cyclin A2/E1 activation defines a hepatocellular carcinoma subclass with a rearrangement signature of replication stress. Nat. Commun..

[39] Nwosu Z.C., Megger D.A., Hammad S., Sitek B., Roessler S., Ebert M.P., Meyer C., Dooley S. (2017). Identification of the Consistently Altered Metabolic Targets in Human Hepatocellular Carcinoma. Cell Mol. Gastroenterol. Hepatol..

[40] Macheda M.L., Rogers S., Best J.D. (2005). Molecular and cellular regulation of glucose transporter (GLUT) proteins in cancer. J. Cell Physiol..

[41] Amann T., Maegdefrau U., Hartmann A., Agaimy A., Marienhagen J., Weiss T.S., Stoeltzing O., Warnecke C., Schölmerich J., Oefner P.J. (2009). GLUT1 expression is increased in hepatocellular carcinoma and promotes tumorigenesis. Am. J. Pathol..

[42] Chen H.L., OuYang H.Y., Le Y., Jiang P., Tang H., Yu Z.S., He M.K., Tang Y.Q., Shi M. (2018). Aberrant MCT4 and GLUT1 expression is correlated with early recurrence and poor prognosis of hepatocellular carcinoma after hepatectomy. Cancer Med..

[43] Paudyal B., Paudyal P., Oriuchi N., Tsushima Y., Nakajima T., Endo K. (2008). Clinical Implications of glucose transport and metabolism evaluated by 18F-FDG PET in hepatocellular carcinoma. Int. J. Oncol..

[44] Daskalow K., Pfander D., Weichert W., Rohwer N., Thelen A., Neuhaus P., Jonas S., Wiedenmann B., Benckert C., Cramer T. (2009). Distinct temporospatial expression patterns of glycolysis-related proteins in human hepatocellular carcinoma. Histochem. Cell Biol..

[45] Kim Y.H., Jeong D.C., Pak K., Han M.E., Kim J.Y., Liangwen L., Kim H.J., Kim T.W., Kim T.H., Hyun D.W. (2017). SLC2A2 (GLUT2) as a novel prognostic factor for hepatocellularcarcinoma. Oncotarget.

[46] Gao H., Hao Y., Zhou X., Li H., Liu F., Zhu H., Song X., Niu Z., Ni Q., Chen M.S. (2020). Prognostic value of glucose transporter 3 expression in hepatocellular carcinoma. Oncol. Lett..

[47] Wilson J.E. (2003). Isozymes of mammalian hexokinase: Structure, subcellular localization and metabolic function. J. Exp. Biol..

[48] Mathupala S.P., Rempel A., Pedersen P.L. (1997). Aberrant glycolytic metabolism of cancer cells: A remarkable coordination of genetic, transcriptional, post-translational, and mutational events that lead to a critical role for type II hexokinase. J. Bioenerg. Biomembr..

[49] Pedersen P.L., Mathupala S., Rempel A., Geschwind J.F., Ko Y.H. (2002). Mitochondrial bound type II hexokinase: A key player in the growth and survival of many cancers and an ideal prospect for therapeutic intervention. Biochim. Biophys. Acta.

[50] Guzman G., Chennuri R., Chan A., Rea B., Quintana A., Patel R., Xu P.Z., Xie H., Hay N. (2015). Evidence for heightened hexokinase II immunoexpression in hepatocyte dysplasia and hepatocellular carcinoma. Dig. Dis. Sci..

[51] Gong L., Cui Z., Chen P., Han H., Peng J., Leng X. (2012). Reduced survival of patients with hepatocellular carcinoma expressing hexokinase II. Med. Oncol..

[52] Kwee S.A., Hernandez B., Chan O., Wong L. (2012). Choline kinase alpha and hexokinase-2 protein expression in hepatocellular carcinoma: Association with survival. PLoS ONE.

[53] Hong X., Song R., Song H., Zheng T., Wang J., Liang Y., Qi S., Lu Z., Song X., Jiang H. (2014). PTEN antagonises Tcl1/hnRNPK-mediated G6PD pre-mRNA splicing which contributes to hepatocarcinogenesis. Gut.

[54] Kowalik M.A., Guzzo G., Morandi A., Perra A., Menegon S., Masgras I., Trevisan E., Angioni M.M., Fornari F., Quagliata L. (2016). Metabolic reprogramming identifies the most aggressive lesions at early phases of hepatic carcinogenesis. Oncotarget.

[55] Hu H., Ding X., Yang Y., Zhang H., Li H., Tong S., An X., Zhong Q., Liu X., Ma L. (2014). Changes in glucose-6-phosphate dehydrogenase expression results in altered behavior of HBV-associated liver cancer cells. Am. J. Physiol. Gastrointest. Liver Physiol..

[56] Xu I.M., Lai R.K., Lin S.H., Tse A.P., Chiu D.K., Koh H.Y., Law C.T., Wong C.M., Cai Z., Wong C.C. (2016). Transketolase counteracts oxidative stress to drive cancer development. Proc. Natl. Acad. Sci. USA.

[57] Liu A.M., Xu Z., Shek F.H., Wong K.F., Lee N.P., Poon R.T., Chen J., Luk J.M. (2014). miR-122 targets pyruvate kinase M2 and affects metabolism of hepatocellular carcinoma. PLoS ONE.

[58] Chen Z., Lu X., Wang Z., Jin G., Wang Q., Chen D., Chen T., Li J., Fan J., Cong W. (2015). Co-expression of PKM2 and TRIM35 predicts survival and recurrence in hepatocellular carcinoma. Oncotarget.

[59] Wright T.L. (1991). Regenerating nodules—are they premalignant lesions?. Hepatology.

[60] Pinter M., Trauner M., Peck-Radosavljevic M., Sieghart W. (2016). Cancer and liver cirrhosis: Implications on prognosis and management. ESMO Open.

[61] Lee N.C.W., Carella M.A., Papa S., Bubici C. (2018). High Expression of Glycolytic Genes in Cirrhosis Correlates with the Risk of Developing Liver Cancer. Front. Cell Dev. Biol..

[62] Nwosu Z.C., Battello N., Rothley M., Piorońska W., Sitek B., Ebert M.P., Hofmann U., Sleeman J., Wölfl S., Meyer C. (2018). Liver cancer cell lines distinctly mimic the metabolic gene expression pattern of the corresponding human tumours. J. Exp. Clin. Cancer Res..

[63] De Minicis S., Kisseleva T., Francis H., Baroni G.S., Benedetti A., Brenner D., Alvaro D., Alpini G., Marzioni M. (2013). Liver carcinogenesis: Rodent models of hepatocarcinoma and cholangiocarcinoma. Dig. Liver Dis..

[64] Santos N.P., Colaço A.A., Oliveira P.A. (2017). Animal models as a tool in hepatocellular carcinoma research: A Review. Tumour Biol..

[65] Vesselinovitch S.D., Mihailovich N. (1983). Kinetics of diethylnitrosamine hepatocarcinogenesis in the infant mouse. Cancer Res..

[66] Klimek F., Mayer D., Bannasch P. (1984). Biochemical microanalysis of glycogen content and glucose-6-phosphate dehydrogenase activity in focal lesions of the rat liver induced by N-nitrosomorpholine. Carcinogenesis.

[67] Bannasch P. (1990). Pathobiology of chemical hepatocarcinogenesis: Recent progress and perspectives. Part II. Metabolic and molecular changes. J. Gastroenterol. Hepatol..

[68] Hacker H.J., Mtiro H., Bannasch P., Vesselinovitch S.D. (1991). Histochemical profile of mouse hepatocellular adenomas and carcinomas induced by a single dose of diethylnitrosamine. Cancer Res..

[69] Baba M., Yamamoto R., Iishi H., Tatsuta M., Wada A. (1989). Role of glucose-6-phosphate dehydrogenase on enhanced proliferation of pre-neoplastic and neoplastic cells in rat liver induced by N-nitrosomorpholine. Int. J. Cancer.

[70] Cortinovis C., Klimek F., Nogueira E. (1991). Rat hepatocarcinogenesis induced by N-nitrosodiethylamine and N-nitrosomorpholine continuously administered at low doses. From basophilic areas of hepatocytes to hepatocellular tumors. Am. J. Pathol..

[71] Stumpf H., Bannasch P. (1994). Overexpression of glucose-6-phosphate-dehydrogenase in rat hepatic preneoplasia and neoplasia. Int. J. Oncol..

[72] Moore M.A., Tsuda H., Ito N. (1986). Dehydrogenase histochemistry of N-ethyl-N- hydroxyethylnitrosamine-induced focal liver lesions in the rat—increase in NADPH-generating capacity. Carcinogenesis.

[73] O’Connor P.J. (1981). Interaction of chemical carcinogens with macromolecules. J. Cancer Res. Clin. Oncol..

[74] Cameron R., Farber E. (1981). Some conclusions derived from a liver model for carcinogenesis. Natl. Cancer Inst. Monogr..

[75] Tsuda H., Farber E. (1980). Resistant hepatocytes as early changes in liver induced by polycyclic aromatic hydrocarbons. Int. J. Cancer..

[76] Magee P.N., Lee K.Y. (1964). Cellular injury and carcinogenesis. Alkylation of ribonucleic acid of rat liver by diethylnitrosamine and n-butylmethylnitrosamine in vivo. Biochem. J..

[77] Verna L., Whysner J., Williams G.M. (1996). N-nitrosodiethylamine mechanistic data and risk assessment: Bioactivation, DNA-adduct formation, mutagenicity, and tumor initiation. Pharmacol. Ther..

[78] Hacker H.J., Moore M.A., Mayer D., Bannasch P. (1982). Correlative histochemistry of some enzymes of carbohydrate metabolism in preneoplastic and neoplastic lesions in the rat liver. Carcinogenesis.

[79] Lee J.S., Chu I.S., Mikaelyan A., Calvisi D.F., Heo J., Reddy J.K., Thorgeirsson S.S. (2004). Application of comparative functional genomics to identify best-fit mouse models to study human cancer. Nat. Genet..

[80] Tolba R., Kraus T., Liedtke C., Schwarz M., Weiskirchen R. (2015). Diethylnitrosamine (DEN)-induced carcinogenic liver injury in mice. Lab. Anim..

[81] Li Z.F., Wang J., Huang C., Zhang S., Yang J., Jiang A., Zhou R., Pan D. (2010). Gas chromatography/time-of-flight mass spectrometry-based metabonomics of hepatocarcinoma in rats with lung metastasis: Elucidation of the metabolic characteristics of hepatocarcinoma at formation and metastasis. Rapid Commun. Mass Spectrom..

[82] Wang J., Zhang S., Li Z., Yang J., Huang C., Liang R., Liu Z., Zhou R. (2011). (1)H-NMR-based metabolomics of tumor tissue for the metabolic characterization of rat hepatocellular carcinoma formation and metastasis. Tumour Biol..

[83] Frederiks W.M., Vizan P., Bosch K.S., Vreeling-Sindelárová H., Boren J., Cascante M. (2008). Elevated activity of the oxidative and non-oxidative pentose phosphate pathway in (pre)neoplastic lesions in rat liver. Int. J. Exp. Pathol..

[84] Orrù C., Szydlowska M., Taguchi K., Zavattari P., Perra A., Yamamoto M., Columbano A. (2018). Genetic inactivation of Nrf2 prevents clonal expansion of initiated cells in a nutritional model of rat hepatocarcinogenesis. J. Hepatol..

[85] Lombardi B., Pani P., Schlunk F.F. (1968). Choline-deficiency fatty liver: Impaired release of hepatic triglycerides. J. Lipid Res..

[86] Koteish A., Mae Diehl A. (2002). Animal models of steatohepatitis. Best Pract. Res. Clin. Gastroenterol..

[87] Shinozuka H., Sells M.A., Katyal S.L., Sell S., Lombardi B. (1979). Effects of a choline-devoid diet on the emergence of gamma-glutamyltranspeptidase-positive foci in the liver of carcinogen-treated rats. Cancer Res..

[88] Yokoyama S., Sells M.A., Reddy T.V., Lombardi B. (1985). Hepatocarcinogenic and promoting action of a choline-devoid diet in the rat. Cancer Res..

[89] Sato K., Satoh K., Tsuchida S., Hatayama I., Shen H., Yokoyama Y., Yamada Y., Tamai K. (1992). Specific expression of glutathione S-transferase Pi forms in (pre)neoplastic tissues: Their properties and functions. Tohoku J. Exp. Med..

[90] Mitsuishi Y., Taguchi K., Kawatani Y., Shibata T., Nukiwa T., Aburatani H., Yamamoto M., Motohashi H. (2012). Nrf2 redirects glucose and glutamine into anabolic pathways in metabolic reprogramming. Cancer Cell.

[91] Mitsuishi Y., Motohashi H., Yamamoto M. (2012). The Keap1-Nrf2 system in cancers: Stress response and anabolic metabolism. Front. Oncol..

[92] Marra M., Sordelli I.M., Lombardi A., Lamberti M., Tarantino L., Giudice A., Stiuso P., Abbruzzese A., Sperlongano R., Accardo M. (2011). Molecular targets and oxidative stress biomarkers in hepatocellular carcinoma: An overview. J. Transl. Med..

[93] Fu Y., Chung F.L. (2018). Oxidative stress and hepatocarcinogenesis. Hepatoma Res..

[94] Menegon S., Columbano A., Giordano S. (2016). The Dual Roles of NRF2 in Cancer. Trends Mol. Med..

[95] Orrù C., Perra A., Kowalik M.A., Rizzolio S., Puliga E., Cabras L., Giordano S., Columbano A. (2020). Distinct Mechanisms Are Responsible for Nrf2-Keap1 Pathway Activation at Different Stages of Rat Hepatocarcinogenesis. Cancers (Basel).

[96] Solt D.B., Medline A., Farber E. (1977). Rapid emergence of carcinogen-induced hyperplastic lesions in a new model for the sequential analysis of liver carcinogenesis. Am. J. Pathol..

[97] Kowalik M.A., Sulas P., Ledda-Columbano G.M., Giordano S., Columbano A., Perra A. (2015). Cytokeratin-19 positivity is acquired along cancer progression and does not predict cell origin in rat hepatocarcinogenesis. Oncotarget.

[98] Lee J.S., Heo J., Libbrecht L., Chu I.S., Kaposi-Novak P., Calvisi D.F., Mikaelyan A., Roberts L.R., Demetris A.J., Sun Z. (2006). A novel prognostic subtype of human hepatocellular carcinoma derived from hepatic progenitor cells. Nat. Med..

[99] Yang X.R., Xu Y., Yu B., Zhou J., Qiu S.J., Shi G.M., Zhang B.H., Wu W.Z., Shi Y.H., Wu B. (2010). High expression levels of putative hepatic stem/progenitor cell biomarkers related to tumour angiogenesis and poor prognosis of hepatocellular carcinoma. Gut.

[100] Andersen J.B., Loi R., Perra A., Factor V.M., Ledda-Columbano G.M., Columbano A., Thorgeirsson S.S. (2010). Progenitor-derived hepatocellular carcinoma model in the rat. Hepatology.

[101] Sciacovelli M., Guzzo G., Morello V., Frezza C., Zheng L., Nannini N., Calabrese F., Laudiero G., Esposito F., Landriscina M. (2013). The mitochondrial chaperone TRAP1 promotes neoplastic growth by inhibiting succinate dehydrogenase. Cell Metab..

[102] Rasola A., Neckers L., Picard D. (2014). Mitochondrial oxidative phosphorylation TRAP(1)ped in tumor cells. Trends Cell Biol..

[103] Guzzo G., Sciacovelli M., Bernardi P., Rasola A. (2014). Inhibition of succinate dehydrogenase by the mitochondrial chaperone TRAP1 has anti-oxidant and anti-apoptotic effects on tumor cells. Oncotarget.

[104] Semenza G.L. (1998). Hypoxia-inducible factor 1: Master regulator of O2 homeostasis. Curr. Opin. Genet. Dev..

[105] Icard P., Poulain L., Lincet H. (2012). Understanding the central role of citrate in the metabolism of cancer cells. Biochim. Biophys. Acta.

[106] Hayashi Y. (1992). Overview of genotoxic carcinogens and non-genotoxic carcinogens. Exp. Toxicol. Pathol..

[107] Williams G.M. (1997). Chemicals with carcinogenic activity in the rodent liver; mechanistic evaluation of human risk. Cancer Lett..

[108] Lee S.J., Yum Y.N., Kim S.C., Kim Y., Lim J., Lee W.J., Koo K.H., Kim J.H., Kim J.E., Lee W.S. (2013). Distinguishing between genotoxic and non-genotoxic hepatocarcinogens by gene expression profiling and bioinformatic pathway analysis. Sci. Rep..

[109] Diwan B.A., Lubet R.A., Ward J.M., Hrabie J.A., Rice J.M. (1992). Tumor-promoting and hepatocarcinogenic effects of 1,4-bis[2-(3,5-dichloropyridyloxy)]benzene (TCPOBOP) in DBA/2NCr and C57BL/6NCr mice and an apparent promoting effect on nasal cavity tumors but not on hepatocellular tumors in F344/NCr rats initiated with N-nitrosodiethylamine. Carcinogenesis.

[110] Tzameli I., Pissios P., Schuetz E.G., Moore D.D. (2000). The xenobiotic compound 1,4-bis[2-(3,5-dichloropyridyloxy)]benzene is an agonist ligand for the nuclear receptor CAR. Mol. Cell Biol..

[111] Rao M.S., Tatematsu M., Subbarao V., Ito N., Reddy J.K. (1986). Analysis of peroxisome proliferator-induced preneoplastic and neoplastic lesions of rat liver for placental form of glutathione S-transferase and gamma-glutamyltranspeptidase. Cancer Res..

[112] Rao M.S., Subbarao V., Kumar S., Yeldandi A.V., Reddy J.K. (1992). Phenotypic properties of liver tumors induced by dehydroepiandrosterone in F-344 rats. Jpn. J. Cancer Res..

[113] Greaves P., Irisarri E., Monro A.M. (1986). Hepatic foci of cellular and enzymatic alteration and nodules in rats treated with clofibrate or diethylnitrosamine followed by phenobarbital: Their rate of onset and their reversibility. J. Natl. Cancer Inst..

[114] Rao K.N., Elm M.S., Kelly R.H., Chandar N., Brady E.P., Rao B., Shinozuka H., Eagon P.K. (1997). Hepatic hyperplasia and cancer in rats: Metabolic alterations associated with cell growth. Gastroenterology.

[115] Nishikawa T., Bellance N., Damm A., Bing H., Zhu Z., Handa K., Yovchev M.I., Sehgal V., Moss T.J., Oertel M. (2014). A switch in the source of ATP production and a loss in capacity to perform glycolysis are hallmarks of hepatocyte failure in advance liver disease. J. Hepatol..

[116] Aydinlik H., Nguyen T.D., Moennikes O., Buchmann A., Schwarz M. (2001). Selective pressure during tumor promotion by phenobarbital leads to clonal outgrowth of beta-catenin-mutated mouse liver tumors. Oncogene.

[117] Chafey P., Finzi L., Boisgard R., Caüzac M., Clary G., Broussard C., Pégorier J.P., Guillonneau F., Mayeux P., Camoin L. (2009). Proteomicanalysis of beta-catenin activation in mouse liver by DIGE analysis identifies glucose metabolism as a new target of the Wnt pathway. Proteomics.

[118] Lee S.Y., Jeon H.M., Ju M.K., Kim C.H., Yoon G., Han S.I., Park H.G., Kang H.S. (2012). Wnt/Snail signaling regulates cytochrome C oxidase and glucose metabolism. Cancer Res..

[119] Unterberger E.B., Eichner J., Wrzodek C., Lempiäinen H., Luisier R., Terranova R., Metzger U., Plummer S., Knorpp T., Braeuning A. (2014). Ha-ras and β-catenin oncoproteins orchestrate metabolic programs in mouse liver tumors. Int. J. Cancer..

[120] Yuneva M.O., Fan T.W., Allen T.D., Higashi R.M., Ferraris D.V., Tsukamoto T., Matés J.M., Alonso F.J., Wang C., Seo Y. (2012). The metabolic profile of tumors depends on both the responsible genetic lesion and tissue type. Cell Metab..

[121] Tward A.D., Jones K.D., Yant S., Cheung S.T., Fan S.T., Chen X., Kay M.A., Wang R., Bishop J.M. (2007). Distinct pathways of genomic progression to benign and malignant tumors of the liver. Proc. Natl. Acad. Sci. USA.

[122] Mattu S., Saliba C., Sulas P., Zavattari P., Perra A., Kowalik M.A., Monga S.P., Columbano A. (2018). High Frequency of β-Catenin Mutations in Mouse Hepatocellular Carcinomas Induced by a Nongenotoxic Constitutive Androstane Receptor Agonist. Am. J. Pathol..

[123] Tong C., Li P., Wu N.L., Yan Y., Ying Q.L. (2010). Production of p53 gene knockout rats by homologous recombination in embryonic stem cells. Nature.

[124] Taguchi K., Takaku M., Egner P.A., Morita M., Kaneko T., Mashimo T., Kensler T.W., Yamamoto M. (2016). Generation of a New Model Rat: Nrf2 Knockout Rats Are Sensitive to Aflatoxin B1 Toxicity. Toxicol. Sci..

[125] Wise D.R., DeBerardinis R.J., Mancuso A., Sayed N., Zhang X.Y., Pfeiffer H.K., Nissim I., Daikhin E., Yudkoff M., McMahon S.B. (2008). Myc regulates a transcriptional program that stimulates mitochondrial glutaminolysis and leads to glutamine addiction. Proc. Natl. Acad. Sci. USA.

[126] Yuneva M. (2008). Finding an “Achilles’ heel” of cancer: The role of glucose and glutamine metabolism in the survival of transformed cells. Cell Cycle.

[127] Fafalios A., Ma J., Tan X., Stoops J., Luo J., Defrances M.C., Zarnegar R. (2011). A hepatocyte growth factor receptor (Met)-insulin receptor hybrid governs hepatic glucose metabolism. Nat. Med..

[128] MacDonald B.T., Tamai K., He X. (2009). Wnt/beta-catenin signaling: Components, mechanisms, and diseases. Dev. Cell..

[129] Monga S.P. (2015). β-Catenin Signaling and Roles in Liver Homeostasis, Injury, and Tumorigenesis. Gastroenterology.

[130] Shachaf C.M., Kopelman A.M., Arvanitis C., Karlsson A., Beer S., Mandl S., Bachmann M.H., Borowsky A.D., Ruebner B., Cardiff R.D. (2004). MYC inactivation uncovers pluripotent differentiation and tumour dormancy in hepatocellular cancer. Nature.

[131] Wang R., Ferrell L.D., Faouzi S., Maher J.J., Bishop J.M. (2001). Activation of the Met receptor by cell attachment induces and sustains hepatocellular carcinomas in transgenic mice. J. Cell Biol..

[132] De Matteis S., Ragusa A., Marisi G., De Domenico S., Casadei Gardini A., Bonafè M., Giudetti A.M. (2018). Aberrant Metabolism in Hepatocellular Carcinoma Provides Diagnostic and Therapeutic Opportunities. Oxid Med. Cell Longev..

[133] Wang H., Lu J., Edmunds L.R., Kulkarni S., Dolezal J., Tao J., Ranganathan S., Jackson L., Fromherz M., Beer-Stolz D. (2016). Coordinated Activities of Multiple Myc-dependent and Myc-independent Biosynthetic Pathways in Hepatoblastoma. J. Biol. Chem..

[134] Dolezal J.M., Wang H., Kulkarni S., Jackson L., Lu J., Ranganathan S., Goetzman E.S., Bharathi S.S., Beezhold K., Byersdorfer C.A. (2017). Sequential adaptive changes in a c-Myc-driven model of hepatocellular carcinoma. J. Biol. Chem..

[135] He F., Antonucci L., Yamachika S., Zhang Z., Taniguchi K., Umemura A., Hatzivassiliou G., Roose-Girma M., Reina-Campos M., Duran A. (2020). NRF2 activates growth factor genes and downstream AKT signaling to induce mouse and human hepatomegaly. J. Hepatol..

[136] Santacatterina F., Sánchez-Cenizo L., Formentini L., Mobasher M.A., Casas E., Rueda C.B., Martínez-Reyes I., Núñez de Arenas C., García-Bermúdez J., Zapata J.M. (2016). Down-regulation of oxidative phosphorylation in the liver by expression of the ATPase inhibitory factor 1 induces a tumor-promoter metabolic state. Oncotarget.

[137] Sánchez-Aragó M., Formentini L., Cuezva J.M. (2013). Mitochondria-mediated energy adaption in cancer: The H(+)-ATP synthase-geared switch of metabolism in human tumors. Antioxid. Redox Signal..

[138] Calvisi D.F., Wang C., Ho C., Ladu S., Lee S.A., Mattu S., Destefanis G., Delogu S., Zimmermann A., Ericsson J. (2011). Increased lipogenesis, induced by AKT-mTORC1-RPS6 signaling, promotes development of human hepatocellular carcinoma. Gastroenterology.

[139] Gatenby R.A., Gillies R.J. (2004). Why do cancers have high aerobic glycolysis?. Nat. Rev. Cancer..

[140] Patra K.C., Wang Q., Bhaskar P.T., Miller L., Wang Z., Wheaton W., Chandel N., Laakso M., Muller W.J., Allen E.L. (2013). Hexokinase 2 is required for tumor initiation and maintenance and its systemic deletion is therapeutic in mouse models of cancer. Cancer Cell.

[141] DeWaal D., Nogueira V., Terry A.R., Patra K.C., Jeon S.M., Guzman G., Au J., Long C.P., Antoniewicz M.R., Hay N. (2018). Hexokinase-2 depletion inhibits glycolysis and induces oxidative phosphorylation in hepatocellular carcinoma and sensitizes to metformin. Nat. Commun..

[142] Dayton T.L., Gocheva V., Miller K.M., Israelsen W.J., Bhutkar A., Clish C.B., Davidson S.M., Luengo A., Bronson R.T., Jacks T. (2016). Germline loss of PKM2 promotes metabolic distress and hepatocellular carcinoma. Genes Dev..

[143] Méndez-Lucas A., Li X., Hu J., Che L., Song X., Jia J., Wang J., Xie C., Driscoll P.C., Tschaharganeh D.F. (2017). Glucose Catabolism in Liver Tumors Induced by c-MYC Can Be Sustained by Various PKM1/PKM2 Ratios and Pyruvate Kinase Activities. Cancer Res..

[144] Ngo H.K.C., Kim D.H., Cha Y.N., Na H.K., Surh Y.J. (2017). Nrf2 Mutagenic Activation Drives Hepatocarcinogenesis. Cancer Res..

[145] Kowalik M.A., Puliga E., Cabras L., Sulas P., Petrelli A., Perra A., Ledda-Columbano G.M., Morandi A., Merlin S., Orrù C. (2020). Thyroid hormone inhibits hepatocellular carcinoma progression via induction of differentiation and metabolic reprogramming. J. Hepatol..

[146] Kudo Y., Sugimoto M., Arias E., Kasashima H., Cordes T., Linares J.F., Duran A., Nakanishi Y., Nakanishi N., L’Hermitte A. (2020). PKCλ/ι Loss Induces Autophagy, Oxidative Phosphorylation, and NRF2 to Promote Liver Cancer Progression. Cancer Cell.

[147] DeBerardinis R.J., Mancuso A., Daikhin E., Nissim I., Yudkoff M., Wehrli S., Thompson C.B. (2007). Beyond aerobic glycolysis: Transformed cells can engage in glutamine metabolism that exceeds the requirement for protein and nucleotide synthesis. Proc. Natl. Acad. Sci. USA.

[148] Carracedo A., Cantley L.C., Pandolfi P.P. (2013). Cancer metabolism: Fatty acid oxidation in the limelight. Nat. Rev. Cancer.

[149] Ma Y., Temkin S.M., Hawkridge A.M., Guo C., Wang W., Wang X.Y., Fang X. (2018). Fatty acid oxidation: An emerging facet of metabolic transformation in cancer. Cancer Lett..

[150] Che L., Pilo M.G., Cigliano A., Latte G., Simile M.M., Ribback S., Dombrowski F., Evert M., Chen X., Calvisi D.F. (2017). Oncogene dependent requirement of fatty acid synthase in hepatocellular carcinoma. Cell Cycle.

[151] Beloribi-Djefaflia S., Vasseur S., Guillaumond F. (2016). Lipid metabolic reprogramming in cancer cells. Oncogenesis.

[152] Hu J., Che L., Li L., Pilo M.G., Cigliano A., Ribback S., Li X., Latte G., Mela M., Evert M. (2016). Co-activation of AKT and c-Met triggers rapid hepatocellular carcinoma development via the mTORC1/FASN pathway in mice. Sci. Rep..

[153] Li L., Pilo G.M., Li X., Cigliano A., Latte G., Che L., Joseph C., Mela M., Wang C., Jiang L. (2016). Inactivation of fatty acid synthase impairs hepatocarcinogenesis driven by AKT in mice and humans. J. Hepatol..

[154] Martínez-Chantar M.L., Vázquez-Chantada M., Ariz U., Martínez N., Varela M., Luka Z., Capdevila A., Rodríguez J., Aransay A.M., Matthiesen R. (2008). Loss of the glycine N-methyltransferase gene leads to steatosis and hepatocellular carcinoma in mice. Hepatology.

[155] Newell P., Villanueva A., Friedman S.L., Koike K., Llovet J.M. (2008). Experimental models of hepatocellular carcinoma. J. Hepatol..

[156] He L., Tian D.A., Li P.Y., He X.X. (2015). Mouse models of liver cancer: Progress and recommendations. Oncotarget.

[157] Fornari F., Gramantieri L., Callegari E., Shankaraiah R.C., Piscaglia F., Negrini M., Giovannini C. (2019). MicroRNAs in Animal Models of HCC. Cancers (Basel).

[158] Goldberg M.S., Sharp P.A. (2012). Pyruvate kinase M2-specific siRNA induces apoptosis and tumor regression. J. Exp. Med..

[159] Pedroza-Torres A., Romero-Córdoba S.L., Justo-Garrido M., Salido-Guadarrama I., Rodríguez-Bautista R., Montaño S., Muñiz-Mendoza R., Arriaga-Canon C., Fragoso-Ontiveros V., Álvarez-Gómez R.M. (2019). MicroRNAs in Tumor Cell Metabolism: Roles and Therapeutic Opportunities. Front. Oncol..

[160] Guo W., Qiu Z., Wang Z., Wang Q., Tan N., Chen T., Chen Z., Huang S., Gu J., Li J. (2015). MiR-199a-5p is negatively associated with malignancies and regulates glycolysis and lactate production by targeting hexokinase 2 in liver cancer. Hepatology.

[161] Xu F., Yan J.J., Gan Y., Chang Y., Wang H.L., He X.X., Zhao Q. (2019). miR-885-5p Negatively Regulates Warburg Effect by Silencing Hexokinase 2 in Liver Cancer. Mol. Ther. Nucleic Acids..

[162] Zhao Y., Li W., Li M., Hu Y., Zhang H., Song G., Yang L., Cai K., Luo Z. (2019). Targeted inhibition of MCT4 disrupts intracellular pH homeostasis and confers self- regulated apoptosis on hepatocellular carcinoma. Exp. Cell Res..

[163] Kroemer G., Pouyssegur J. (2008). Tumor cell metabolism: Cancer’s Achilles’ heel. Cancer Cell.

[164] Brand R.A. (2010). Biographical sketch: Otto Heinrich Warburg, PhD, MD. Clin. Orthop. Relat. Res..

[165] Vander Heiden M.G. (2011). Targeting cancer metabolism: A therapeutic window opens. Nat. Rev. Drug Discov..

[166] Calvisi D.F., Frau M., Tomasi M.L., Feo F., Pascale R.M. (2012). Deregulation of signalling pathways in prognostic subtypes of hepatocellular carcinoma: Novel insights from interspecies comparison. Biochim. Biophys. Acta.

[167] Pelicano H., Martin D.S., Xu R.H., Huang P. (2006). Glycolysis inhibition for anticancer treatment. Oncogene.

[168] Kim W., Yoon J.H., Jeong J.M., Cheon G.J., Lee T.S., Yang J.I., Park S.C., Lee H.S. (2007). Apoptosis-inducing antitumor efficacy of hexokinase II inhibitor in hepatocellular carcinoma. Mol. Cancer Ther..

[169] Pernicova I., Korbonits M. (2014). Metformin--mode of action and clinical implications for diabetes and cancer. Nat. Rev. Endocrinol..

[170] Wang H., Lu J., Dolezal J., Kulkarni S., Zhang W., Chen A., Gorka J., Mandel J.A., Prochownik E.V. (2019). Inhibition of hepatocellular carcinoma by metabolic normalization. PLoS ONE.

[171] Cadoret A., Ovejero C., Terris B., Souil E., Lévy L., Lamers W.H., Kitajewski J., Kahn A., Perret C. (2002). New targets of beta-catenin signaling in the liver are involved in the glutamine metabolism. Oncogene.

[172] Adebayo Michael A.O., Ko S., Tao J., Moghe A., Yang H., Xu M., Russell J.O., Pradhan-Sundd T., Liu S., Singh S. (2019). Inhibiting Glutamine-Dependent mTORC1 Activation Ameliorates Liver Cancers Driven by β-Catenin Mutations. Cell Metab..

[173] Ballou L.M., Lin R.Z. (2008). Rapamycin and mTOR kinase inhibitors. J. Chem. Biol..

[174] Columbano A., Chiellini G., Kowalik M.A. (2017). GC-1: A Thyromimetic With Multiple Therapeutic Applications in Liver Disease. Gene Expr..

[175] Puliga E., Min Q., Tao J., Zhang R., Pradhan-Sundd T., Poddar M., Singh S., Columbano A., Yu J., Monga S.P. (2017). Thyroid Hormone Receptor-β Agonist GC-1 Inhibits Met-β-Catenin-Driven Hepatocellular Cancer. Am. J. Pathol..

[176] Brent G.A. (1994). The molecular basis of thyroid hormone action. N. Engl. J. Med..

[177] Cheng S.Y., Leonard J.L., Davis P.J. (2010). Molecular aspects of thyroid hormone actions. Endocr. Rev..

[178] Weitzel J.M., Iwen K.A. (2011). Coordination of mitochondrial biogenesis by thyroid hormone. Mol. Cell Endocrinol..

[179] Lazar M.A. (1993). Thyroid hormone receptors: Multiple forms, multiple possibilities. Endocr. Rev..

[180] Frau C., Loi R., Petrelli A., Perra A., Menegon S., Kowalik M.A., Pinna S., Leoni V.P., Fornari F., Gramantieri L. (2015). Local hypothyroidism favors the progression of preneoplastic lesions to hepatocellular carcinoma in rats. Hepatology.

[181] Ledda-Columbano G.M., Perra A., Loi R., Shinozuka H., Columbano A. (2000). Cell proliferation induced by triiodothyronine in rat liver is associated with nodule regression and reduction of hepatocellular carcinomas. Cancer Res..

[182] Chi H.C., Chen S.L., Tsai C.Y., Chuang W.Y., Huang Y.H., Tsai M.M., Wu S.M., Sun C.P., Yeh C.T., Lin K.H. (2016). Thyroid hormone suppresses hepatocarcinogenesis via DAPK2 and SQSTM1-dependent selective autophagy. Autophagy.

[183] Chi H.C., Chen S.L., Lin S.L., Tsai C.Y., Chuang W.Y., Lin Y.H., Huang Y.H., Tsai M.M., Yeh C.T., Lin K.H. (2017). Thyroid hormone protects hepatocytes from HBx-induced carcinogenesis by enhancing mitochondrial turnover. Oncogene.

[184] Cioffi F., Senese R., Lanni A., Goglia F. (2013). Thyroid hormones and mitochondria: With a brief look at derivatives and analogues. Mol. Cell Endocrinol..

[185] Sinha R.A., Singh B., Zhou J., Wu Y., Farah B.L., Ohba K., Lesmana R., Gooding J., Bay B.H., Yen P.M. (2015). Thyroid hormone induction of mitochondrial activity is coupled to mitophagy via ROS-AMPK-ULK1 signaling. Autophagy.

[186] Klein I., Ojamaa K. (2001). Thyroid hormone and the cardiovascular system. N. Engl. J. Med..

[187] Marks P.A., Banks J. (1960). Inhibition of mammalian glucose-6-phosphate dehydrogenase by steroids. Proc. Natl. Acad. Sci. USA.

[188] Garcea R., Daino L., Pascale R., Frassetto S., Cozzolino P., Ruggiu M.E., Feo F. (1987). Inhibition by dehydroepiandrosterone of liver preneoplastic foci formation in rats after initiation-selection in experimental carcinogenesis. Toxicol. Pathol..

[189] Simile M., Pascale R.M., De Miglio M.R., Nufris A., Daino L., Seddaiu M.A., Muroni M.R., Rao K.N., Feo F. (1995). Inhibition by dehydroepiandrosterone of growth and progression of persistent liver nodules in experimental rat liver carcinogenesis. Int. J. Cancer.

[190] Ghergurovich J.M., García-Cañaveras J.C., Wang J., Schmidt E., Zhang Z., TeSlaa T., Patel H., Chen L., Britt E.C., Piqueras-Nebot M. (2020). A small molecule G6PD inhibitor reveals immune dependence on pentose phosphate pathway. Nat. Chem. Biol..

[191] Petrelli A., Perra A., Cora D., Sulas P., Menegon S., Manca C., Migliore C., Kowalik M.A., Ledda-Columbano G.M., Giordano S. (2014). MicroRNA/gene profiling unveils early molecular changes and nuclear factor erythroid related factor 2 (NRF2) activation in a rat model recapitulating human hepatocellular carcinoma (HCC). Hepatology.

